# One-pot enzymatic synthesis of the sugar nucleotide CDP–ribitol

**DOI:** 10.1039/d6cb00172f

**Published:** 2026-09-09

**Authors:** Saeed Akkad, Eva W. Wan, Alex Maddison, Jessica Rule Mcloughlin, Cindy Au-Yeung, Lloyd D. Murphy, Greg L. McNeil, Lianne I. Willems

**Affiliations:** a York Structural Biology Laboratory and York Biomedical Research Institute, Department of Chemistry, University of York York YO10 5DD UK lianne.willems@york.ac.uk; b Department of Chemistry, University of York York YO10 5DD UK

## Abstract

CDP–ribitol serves as an essential donor for d-ribitol–5-phosphate incorporation into bacterial and mammalian glycoconjugates. Here, we demonstrate the one-pot enzymatic synthesis of CDP–ribitol from the readily available precursor ribitol. We also explore the synthesis of bioorthogonally tagged derivatives and other CDP conjugates, thereby providing valuable new tools for glycoscience research.

## Abstract 2

A new approach is described for the enzymatic synthesis of the sugar nucleotide CDP–ribitol on a multi-milligram scale. CDP conjugates are synthesised from readily available polyol precursors *via* a one-pot phosphorylation-cytidylyl transfer cascade.

## Graphical abstract

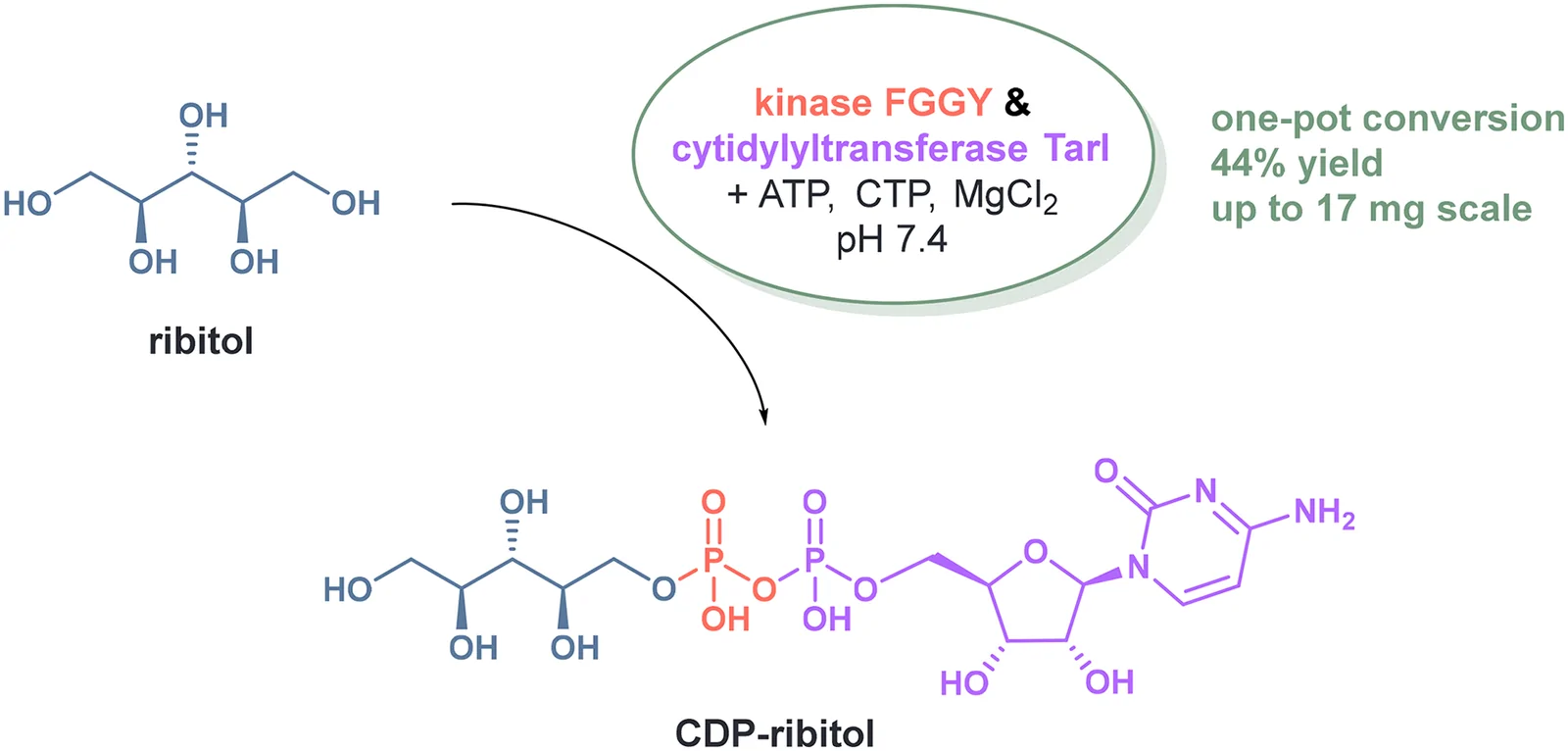

d-Ribitol–5-phosphate (RboP) is a pentitol phosphate that is an integral component of glycopolymers in both bacterial and mammalian systems. In Gram-positive bacteria, RboP is assembled into polymeric chains named wall teichoic acids (WTAs), anionic cell surface glycopolymers that are anchored to the peptidoglycan layer and contribute approximately half the cell wall mass. Together with lipoteichoic acids (LTAs), WTAs play important roles in cell division and cell shape. More importantly, they play a crucial role in the virulence, cell adhesion, biofilm formation and antibiotic susceptibility of several medically relevant bacteria, including *Staphylococcus aureus* and *Streptococcus pneumoniae*.^1–3^ In some Gram-negative bacteria, such as the pathogen *Haemophilus influenzae* type b, the capsular polysaccharide that is responsible for virulence is comprised of repeating units of β-d-ribose–d-ribitol–5-phosphate.^4^ Interestingly, RboP was not known to exist in mammals until only a decade ago, when it was found to be present in a single O-linked glycan on the cell surface glycoprotein α-dystroglycan (α-DG), which acts as a receptor for laminins and other extracellular matrix proteins and is essential in maintaining membrane integrity.^5–8^ Defects in the biosynthesis of this glycan, including the incorporation of RboP, have been associated with an array of muscular dystrophies known collectively as α-dystroglycanopathies, which are also associated with defects of the eye and brain functions.^9,10^

In each of these systems, RboP is transferred onto the acceptor glycan by ribitol–5-phosphate transferases which use the nucleotide-activated form of ribitol, cytidine diphosphate (CDP)–ribitol (Scheme 1), as a universal donor.^7,8,11,12^ Access to CDP–ribitol is essential for further research into the assembly of RboP-containing glycoconjugates and how these can be manipulated for (bio)medical benefit. For example, because of the essential roles WTAs play in virulence and in interacting with the immune system in *S. aureus*-mediated disease, there is significant interest in these glycopolymers as a target for novel antimicrobial agents and as vaccine antigens.^13,14^ In addition, supplementation with CDP–ribitol is being investigated as a strategy to fight aberrant glycosylation in α-dystroglycanopathies with RboP deficiency.^15^ CDP–ribitol is however not commercially available. Therefore, facile methods for the synthesis and isolation of CDP–ribitol on a preparative scale are highly desirable.

**Scheme 1:**
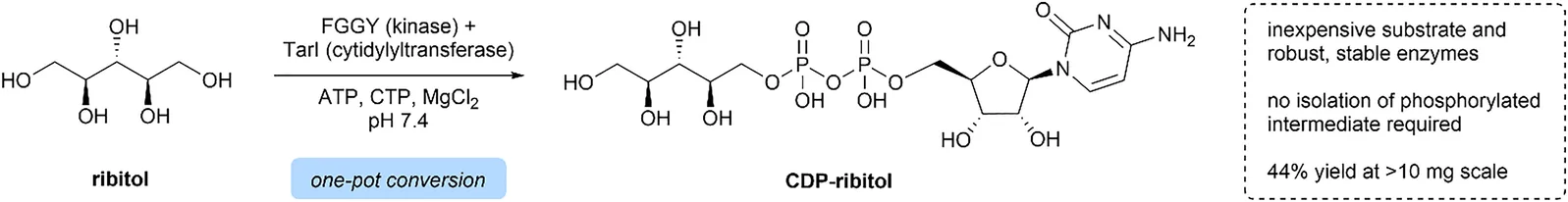
Enzymatic synthesis of CDP–ribitol *via* the one-pot conversion of the pentitol sugar ribitol using the human kinase FGGY and the cytidylyltransferase TarI from *S. aureus*.

Chemical synthesis of CDP–ribitol has been reported in two steps from d-ribose–5-phosphate^16^ or in seven steps from d-ribose,^8^ both involving a key P(v)–P(v) coupling step to generate CDP–ribose or CDP–ribitol. Because some of these reactions and the purification of phosphorylated intermediates can be challenging, enzymatic synthesis offers an attractive alternative. Several enzymatic and chemoenzymatic procedures have been reported,^5,17–20^ which use cytidylyltransferases from bacterial or human origin (*e.g. S. aureus* TarI,^18^*H. influenzae* Bcs1,^21^ or human ISPD)^5,7^ to produce CDP–ribitol from RboP and CTP. The RboP substrate for these enzymatic reactions is not commercially available. It is usually chemically synthesised by reduction of d-ribose–5-phosphate, but its purification can be laborious.^5^

As part of our ongoing research into the labelling of RboP-containing glycans,^22^ we were interested in developing an efficient method for CDP–ribitol synthesis that would avoid the need to isolate RboP. To achieve this, we designed a novel enzymatic strategy from the readily available non-phosphorylated pentitol sugar, ribitol. It is well established that ribitol is not a precursor for CDP–ribitol biosynthesis in bacteria. Instead, the donor is produced by reduction of d-ribulose–5-phosphate, originating from the pentose phosphate pathway,^23^ to RboP and subsequent cytidylyl transfer to generate CDP–ribitol.^18,21^ Mammals synthesise CDP–ribitol through a similar cytidylyl transfer reaction.^5,7^ However, since mammalian cells do not have ribulose–phosphate reductase activity, the metabolic origin of RboP has long remained elusive. Recent evidence revealed it can be formed by the reduction of d-ribose–5-phosphate or alternatively *via* the reduction of d-ribose to ribitol, prior to phosphorylation.^24^ Notably, the latter pathway suggests the existence of a kinase activity to convert ribitol into RboP. Indeed, the human d-ribulose kinase FGGY was shown to display some activity on ribitol *in vitro.*^25^ Intrigued by the possibility of using this kinase as a strategy for RboP production *in vitro*, we set out to develop a one-pot enzymatic approach using FGGY together with the bacterial cytidylyltransferase TarI for the synthesis of CDP–ribitol (Scheme 1). Here, we describe the successful implementation of this novel methodology and demonstrate the isolation of the product CDP–ribitol on a multi-milligram scale. We also assess the promiscuity of the enzymatic system to synthesise unnatural, tagged derivatives of CDP–ribitol as potential bioorthogonal probes and expand its scope towards other biologically relevant CDP conjugates.

## Results and discussion

His-tagged human FGGY and His-tagged TarI from *S. aureus* were readily produced by heterologous expression in an *E. coli* BL21 (DE3) expression system (see SI for experimental procedures). The recombinant enzymes were purified by immobilised metal affinity chromatography and their activity was confirmed by monitoring product formation by LC-MS. Incubation of FGGY with ribitol and ATP led to the formation of a product at *m*/*z* 230.80 ([M–H]^−^), consistent with the mass of RboP and thus confirming the ability of the enzyme to phosphorylate ribitol (Fig. S1).^25^ Additionally, using a pure sample of chemically synthesised RboP^5^ and CTP, TarI was shown to produce the expected CDP–ribitol product (*m*/*z* 536.06 ([M–H]^−^)) (Fig. S2). We did not observe any loss in activity of either enzyme after storage of the purified aliquots for 12 months at −40 °C, suggesting the proteins are stable during long term storage.

**Fig. 1:**
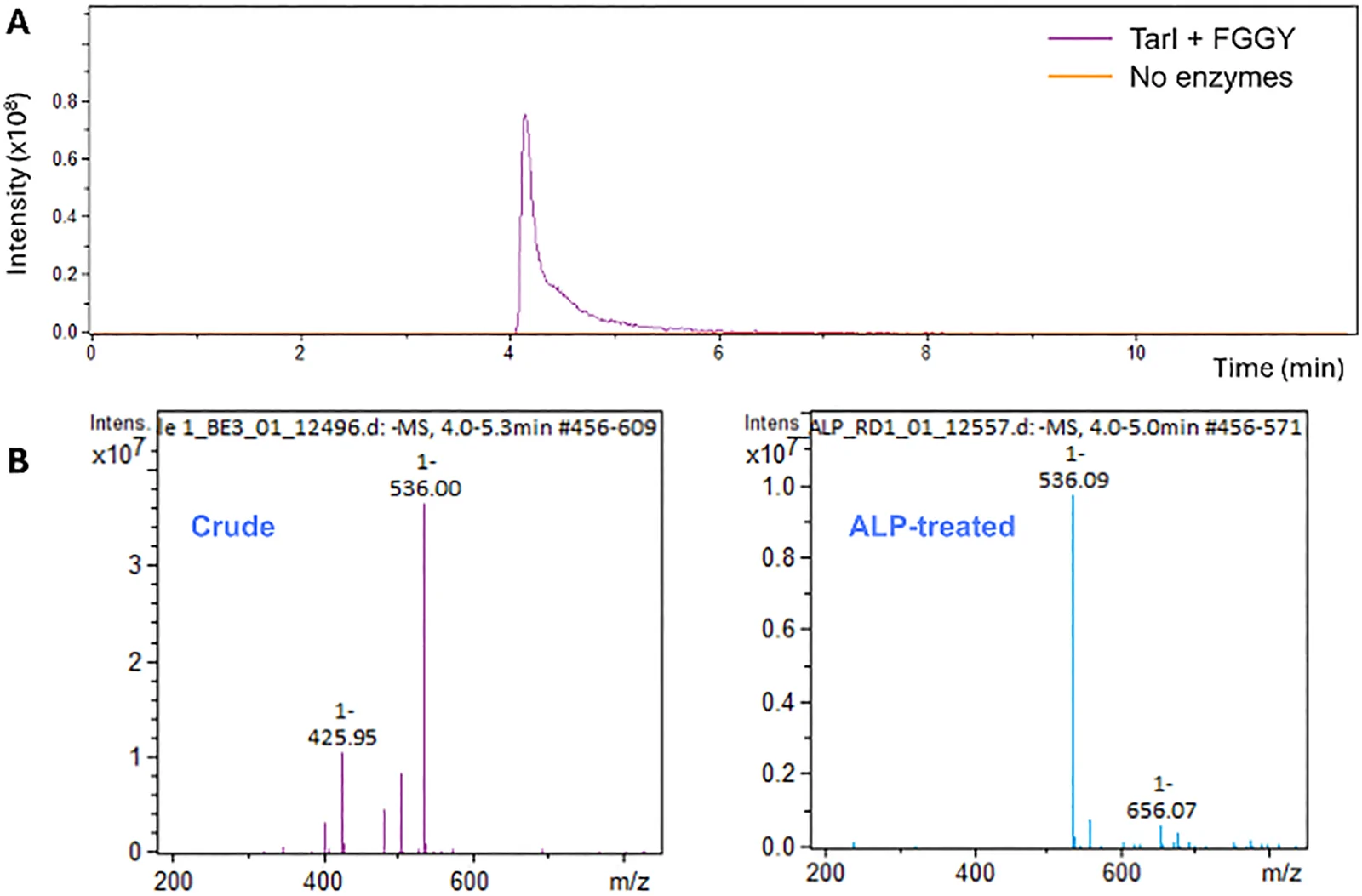
LC-MS analysis of CDP–ribitol formation after overnight incubation of ribitol with recombinant human FGGY and *S. aureus* TarI. (A) Extracted ion chromatogram of CDP–ribitol (purple) compared to a non-enzyme treated control (orange). (B) Mass spectrum of the enzyme-treated sample in (A) before and after ALP treatment, showing the formation of CDP–ribitol (*m*/*z* 536.09, calculated 536.07 ([M–H]^−^)).

Next, the two enzymatic reactions were combined to synthesise CDP–ribitol directly from ribitol. Ribitol was incubated with both enzymes along with ATP and CTP, after which the mixture was treated with alkaline phosphatase (ALP) to dephosphorylate the free nucleotides,^5,7^ which interfered with the detection of CDP–ribitol in our LC-MS method due to peak overlap. Incidentally, ALP treatment also removes any remaining RboP intermediate, simplifying downstream purification.^17^ Upon quenching the reaction by heat inactivation, LC-MS analysis revealed a peak corresponding to CDP–ribitol, demonstrating that both enzymes acted consecutively to convert ribitol into the CDP-activated donor (Fig. 1). The product was absent in control samples where one or both enzymes were omitted.

Having shown that the two reactions successfully proceeded in one pot, we explored the utility of the new method to produce CDP–ribitol on a preparative scale. Hence, multiple 1 mL reactions, each containing 10 µmol of ribitol, were performed in parallel, ALP-treated, quenched and then pooled. The product was purified by anion exchange purification followed by reverse phase preparative HPLC to give pure CDP–ribitol, as confirmed by MS and NMR analysis (Fig. S3A and NMR spectra in the SI, with a yield of 17 mg (44%).

Because issues with CDP–ribitol instability are described in the literature,^12,16,20^ we assessed the stability of the product in buffers of pH 4.5, 7.5 and 9.0 at 20, 37 or 60 °C overnight (Fig. S3B). Apart from small fluctuations in the concentration of the nucleotide sugar, there was no consistent trend that would suggest significant loss of the product under the conditions tested. Additionally, no notable degradation of the purified material was observed during long-term storage at −20 °C. Thus, our approach enables the efficient production of CDP–ribitol on a multi-milligram scale without any significant stability issues.

**Fig. 2:**
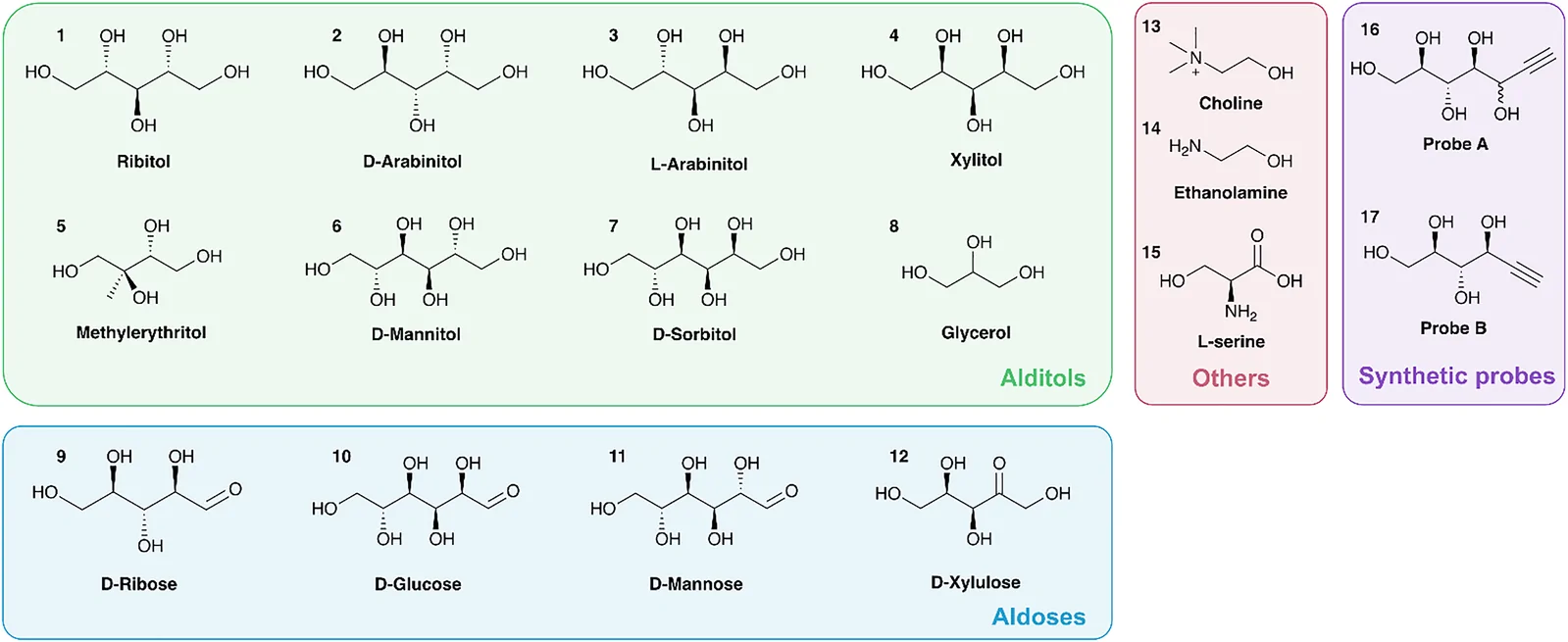
Structures of the substrates tested in the one-pot enzymatic reactions with FGGY/TarI and FGGY/PCYT2.

Encouraged by the efficiency and the scalability of the method, we next explored its substrate flexibility for the synthesis of structurally similar analogues and other biologically relevant CDP conjugates. Small-scale reactions were performed using various substrates (Fig. 2) and their conversion into the corresponding CDP conjugates was monitored by LC-MS analysis (Table 1). Due to the lack of pure standards for most of the expected products, product formation could not be quantified. To provide a qualitative assessment of reaction efficiency, we monitored the intensity of the product's extracted ion chromatogram (EIC) relative to the EIC intensities of other reaction components (*i.e.* CTP), using the formation of CDP–ribitol as a benchmark for good conversion (represented by ‘(+)’ in Table 1). For reactions that generated product peaks of significantly lower intensity, conversion is given as low (±), with the caveat that this approach does not account for differences in ionisation efficiency and does not accurately reflect yields. ALP treatment was not performed in these experiments unless free nucleotides were found to hinder detection of the product.

**Table 1:** Synthesis of CDP-conjugates of various substrates by the enzyme pairs FGGY/TarI and FGGY/PCYT2^a^

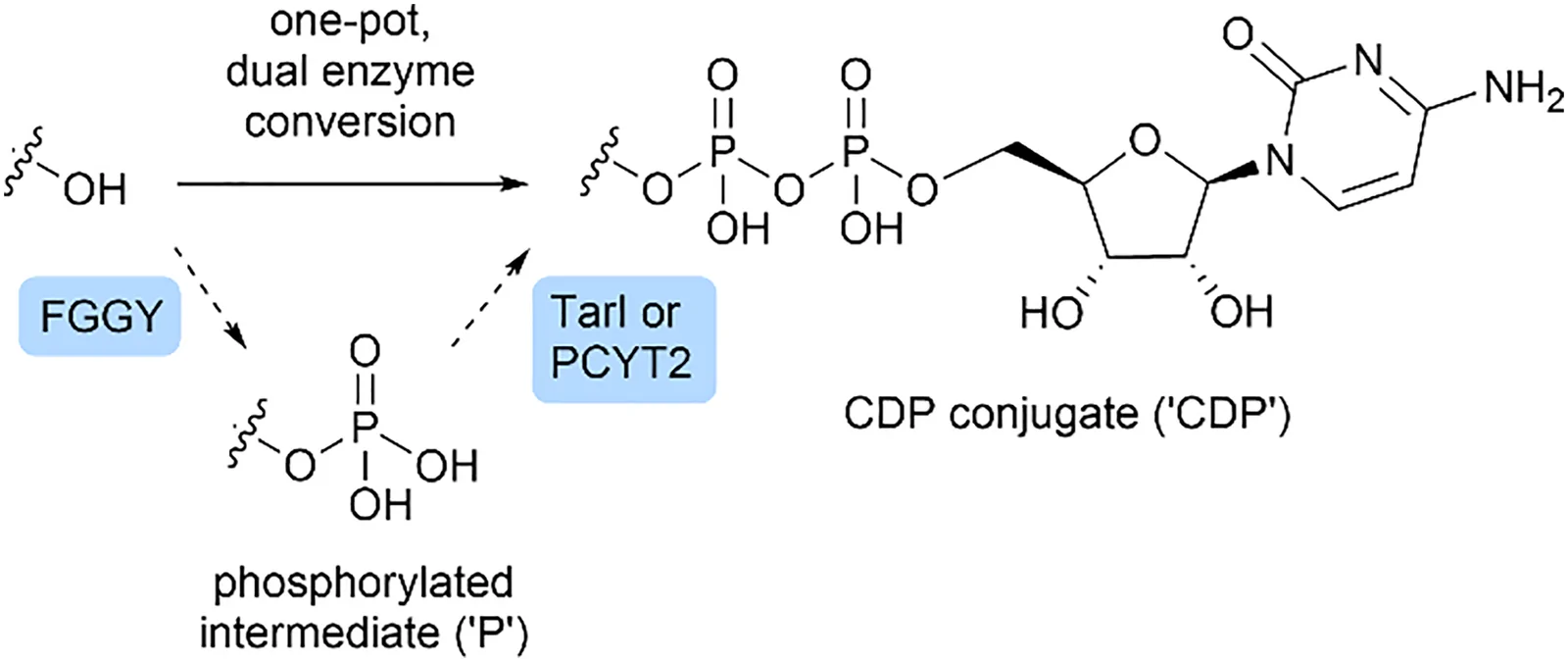		
Substrate	Product formation^b^	
	FGGY/TarI (level of conversion)	FGGY/PCYT2 (level of conversion)
Ribitol 1	CDP (+)	CDP (±)
Probe A 16	CDP (±)^c^	—
Probe B 17	P (+)	—
Alditol sugars		
d-Arabinitol 2	CDP (+)	P (+)
l-Arabinitol 3	CDP (+)	P (+)
Xylitol 4	—	—
Methylerythritol 5	CDP (±)^c^	—
d-Mannitol 6	—	—
d-Sorbitol 7	P (±)	—
Glycerol 8	P (+)	CDP (±)^c^
Aldose sugars		
d-Ribose 9	—	—
d-Glucose 10	—	—
d-Mannose 11	—	—
d-Xylulose 12	CDP (±)^c^	—
Others		
Choline 13	—	—
Ethanolamine 14	—	—
l-Serine 15	—	—

a Standard reaction conditions: 10 mM of substrate (or 5% of glycerol), 5 µM of each recombinant enzyme (FGGY and TarI or FGGY and PCYT2), and 25 mM of CTP, ATP and MgCl_2_ at pH 7.4 overnight.

b Product formation was qualitatively assessed by comparing the product's EIC intensity to that of other reaction components, where (+) denotes good conversion, and (±) denotes a low level of conversion.

c Product formation was only detected when elevated substrate concentrations were used (50 or 200 mM for most substrates, or 20% of glycerol).

Of the alditol sugars tested, both d-arabinitol 2 and l-arabinitol 3 showed conversion into their respective CDP conjugates (Table 1, Fig. S4). d-Arabinitol-1-phosphate is incorporated into the capsular polysaccharide of some serotypes of *S. pneumoniae* to evade the host immune response, with CDP–d-arabinitol acting as the donor.^26,27^ Interestingly, xylitol 4 did not show any conversion, despite its structural similarity to both ribitol and arabinitol. This suggests the precise spatial relationship between the hydroxyl groups is important in ensuring a productive fit in the active site of the enzymes. Interestingly, the branched polyol substrate 2*C*-methyl-d-erythritol 5 was converted into CDP–methylerythritol, although conversion levels were low and a higher substrate concentration was required for detectable product formation compared to ribitol (Table 1, Fig. S5). CDP–methylerythritol is an important intermediate in the 2*C*-methylerythritol 4-phosphate (MEP) pathway which is linked to the biosynthesis of isoprenoids in plants, algae, and bacteria. There is significant interest in its synthesis for the purpose of enzyme discovery, as a precursor for isoprenoid-derived natural products and as a target for novel antimicrobials.^28,29^ While our method was able to produce CDP-activated methylerythritol, the small scale of these experiments prevented confirmation of the position at which the CDP group is attached to the polyol and therefore the exact structure of the product formed from 5 (and other substrates) remains to be determined.

Upon testing further alditol and aldose substrates (Table 1), we did not detect the formation of CDP conjugates of d-mannitol 6 (used in capsular polysaccharide biosynthesis),^30^d-sorbitol 7, d-ribose 9, d-glucose 10 (an intermediate in the biosynthesis of rare dideoxyhexoses for lipopolysaccharide biosynthesis)^31^ or d-mannose 11. FGGY was previously shown to phosphorylate d-ribose at similar levels to d-xylulose,^25^ suggesting that the lack of nucleotide product formation from ribose might be due to TarI being unable to use ribose–phosphate as a substrate. However, we could not detect the formation of ribose–phosphate by LC-MS. Surprisingly, d-xylulose 12 was converted into CDP–d-xylulose, a biosynthetic precursor to CDP–d-arabinitol in *S. pneumoniae*,^27^ when elevated substrate concentrations were used (Fig. S6).

The FGGY/TarI system was unable to convert the smaller substrates glycerol 8, choline 13, ethanolamine 14 and l-serine 15 into their CDP-activated derivatives (Table 1). CDP–choline and CDP–ethanolamine are intermediates in the biosynthesis of membrane phospholipids.^32^ CDP–glycerol is used for the biosynthesis of glycopolymers in both Gram-positive and Gram-negative bacteria, enabling the incorporation of *sn*-glycerol-3-phosphate into WTAs and capsular polysaccharides.^2,33^ While the former two CDP conjugates are commercially available, CDP–glycerol is not. Access to this donor is important to facilitate enzyme discovery and the development of capsular polysaccharide-based vaccines.^33,34^ Enzymatic synthesis of CDP–glycerol has been reported from glycerol–phosphate^34,35^ or glycerol^36^ using the glycerol–phosphate cytidylyltransferases Cps3B/Cps7B or TagD along with the glycerol kinase GlpK. We found that the FGGY/TarI coupled enzyme reaction produced a phosphorylated intermediate, indicating that FGGY was able to act on glycerol as a substrate. However, the CDP-activated donor was not detected, which is perhaps unsurprising given that *S. aureus* expresses distinct cytidylyltransferases to produce CDP–glycerol and CDP–ribitol (TarD and TarI, respectively). Given the biological importance of CDP–glycerol, we expanded our toolkit with the recently identified human cytidylyltransferase PCYT2, which is involved in the biosynthesis of the phospholipid phosphatidyl–ethanolamine but also shows activity on glycerol–phosphate.^37^ His-tagged PCYT2 was expressed and purified as above and then used in combination with FGGY for the one-pot conversion of glycerol. Although the efficiency was low, this reaction successfully generated the expected CDP–glycerol product at *m*/*z* 476.15 ([M–H]^−^) (Table 1, Fig. S7). Interestingly, the same enzyme combination was also able to generate CDP–ribitol, albeit at lower levels than the reaction containing FGGY and TarI (spectra not shown).

We have recently reported the metabolic labelling of O-glycans on the human cell–surface protein α-dystroglycan with the use of alkyne-tagged RboP derivatives as glycan labelling probes.^22^ Because metabolic conversion of these probes into the corresponding CDP-activated donors is required for successful glycosylation, we were interested in exploring the synthesis of the bioorthogonally tagged CDP–ribitol derivatives using our new dual enzyme system. Therefore, two alkyne-tagged ribitol derivatives (probe A (16) and probe B (17)) were used as substrates in the one-pot dual enzyme reaction (Table 1). The CDP conjugate of probe B could not be detected, although a peak corresponding to the phosphorylated intermediate was observed (Fig. S8), suggesting the kinase displays higher substrate flexibility in the region around the C1–OH (numbering according to that in d-ribose) than the cytidylyltransferase. Pleasingly, conversion of probe A into its CDP-activated derivative was successfully detected when the reaction was performed at high substrate concentration (Fig. S9). Upon further optimisation, the ability to produce bioorthogonally tagged CDP–ribitol derivatives may provide an interesting future avenue for the labelling of RboP-containing glycans that avoids the need for cellular donor production and thereby potentially improves labelling efficiency compared to the use of alkyne-tagged RboP-derived probes.^22^ Although the nucleotide may cause issues with cell permeability, a previous study has shown that CDP–ribitol can be taken up by cells and enter the glycosylation pathway, supporting the feasibility of such a labelling strategy.^15^

## Conclusions

This work describes a novel strategy for the enzymatic synthesis of several biologically important CDP conjugates. We have demonstrated the efficient synthesis of CDP–ribitol *via* a dual-enzyme, one-pot conversion of the pentitol sugar, ribitol. Key to the success of our approach was the use of the human ribulokinase FGGY, which displays remarkable substrate flexibility. Coupled with the bacterial cytidylyltransferase TarI, our approach enables access to pure CDP–ribitol in multi-milligram quantities without specialist chemical synthesis expertise. Ribitol is readily available and cheaper than the phosphorylated sugars that are commonly used as CDP–ribitol precursors (ribitol and d-ribose–5-phosphate currently being priced around £1.50 and £100 per gram, respectively). This cost reduction is offset by the need to recombinantly produce an additional protein as compared to existing chemo-enzymatic procedures, in which a single enzymatic step follows the chemical reduction of ribose–phosphate.^38^ However, further optimisation of our enzymatic workflow, for example by implementing repetitive-batch-mode technology^39^ and/or cascade reactions for *in situ* nucleotide formation and regeneration,^36,40^ are likely to enable cost reduction while also paving the way towards larger (gram) scale synthesis.

The enormous diversity in bacterial glycoconjugates means that many of the underlying biosynthetic pathways remain elusive. Efficient methods for the synthesis of sugar nucleotides and their derivatives as chemical tools are essential to accelerate research into glycan biosynthesis and structure and the characterisation of novel enzyme activities. For example, a recent study identified a new RboP transferase responsible for capsule biosynthesis in *Haemophilus influenzae*, requiring access to CDP–ribitol to biochemically characterise the enzyme's activity.^20^ The work presented here also benefits research into the development of RboP transferase inhibitors and offers the potential to accelerate emerging therapeutic approaches, such as CDP–ribitol supplementation for the treatment of α-dystroglycanopathies, the development of WTA biosynthesis inhibitors as novel antimicrobials, and WTA-based synthetic glycoconjugate vaccines.^13–15^ Finally, the flexibility of the FGGY/TarI pair in generating bioorthogonally tagged CDP–ribitol derivatives creates new opportunities in glycan engineering, expanding the available toolkit for glycoscience research.

## Author contributions

S. A. investigation; methodology; validation; visualisation; data curation; writing – original draft. E. W. W., A. M., J. R. M. and C. A.-Y. investigation. L. D. M. and G. L. M. resources. L. I. W. conceptualisation; funding acquisition; writing – review & editing; visualisation; supervision; project administration.

## Conflicts of interest

There are no conflicts to declare.

## Supplementary Material

CB-OLF-D6CB00172F-s001

## Data Availability

Data associated with this publication, including unprocessed NMR spectra and LC-MS data, are available for download from the research data repository of the University of York (DOI: https://doi.org/10.15124/97e507dd-1ead-4c4e-934c-2d9c3aa7d170). Ref. 41 is cited in the supplementary information (SI). Supplementary information available: experimental procedures, supporting figures, and NMR spectra of CDP–ribitol and new compounds. See DOI: https://doi.org/10.1039/d6cb00172f.
