# Supplementary material for: One-pot enzymatic synthesis of the sugar nucleotide CDP–ribitol
Source: RSC Chem Biol. 2026 Sep 9;7(10):1972–7. doi: 10.1039/d6cb00172f (PMC13576159; doi:10.1039/d6cb00172f)
Supplement: Supplementary file 1 [file CB-007-D6CB00172F-s001.pdf]

Supplementary Information (SI) for RSC Chemical Biology.
This journal is © The Royal Society of Chemistry 2026

One-pot enzymatic synthesis of the sugar nucleotide CDP-ribitol

Saeed Akkad, Eva W. Wan, Alex Maddison, Jessica Rule Mcloughlin, Cindy Au-Yeung, Lloyd D. Murphy, Greg L. McNeil and Lianne I. Willems

Supplementary information

Experimental procedures 2
General methods 2
Chemical synthesis of ribitol-5-phosphate (RboP) barium salt 2
Synthesis of 1-ethynyl-D-ribitol (Probe A, compound 16) 3
Synthesis of (2S,3S,4R)-2,3,4,5-tetrahydroxy-pent-1,6-yne (Probe B, compound 17) 3
Expression and purification of recombinant enzymes 4
Enzymatic synthesis of CDP-ribitol 4
General method for the enzymatic synthesis of CDP-derivatives 5
LC-MS analysis of the enzymatic reaction products 6
LC-MS/MS quantitative analysis of CDP-ribitol 6
Supporting figures 7
NMR spectra 12

Experimental procedures

General methods
All reactions were conducted in oven-dried glassware and dry CH_2Cl_2 was freshly distilled. ^{1}H and ^{13}C NMR spectra were recorded on a JEOL ECS400A spectrometer (400 and 101 MHz respectively). Structural assignments were corroborated by homo and heteronuclear 2D NMR methods (COSY, HMQC and DEPT) where necessary. Chemical shifts are reported in parts per million (ppm, δ) relative to the solvent. ^{1}H NMR splitting patterns are designated as singlet (s), doublet (d), triplet (t), quartet (q), doublet of doublets (dd), doublet of doublets of doublets (ddd), doublet of triplets (dt), or multiplet (m). Coupling constants (*J*) are reported in Hertz (Hz). The integrals reported in the ^{1}H NMR peak list for mixtures of diastereomers were normalised so that the numbers of protons for each diastereomer are reported in whole integers. The actual diastereomeric ratio is also given and was determined from the relative integration of the respective proton peaks.

Chemical synthesis of ribitol-5-phosphate (RboP) barium salt

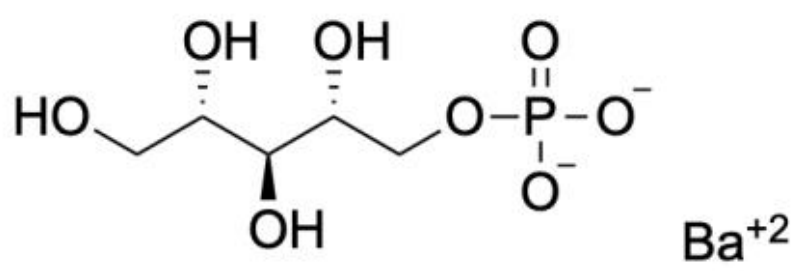

Method adapted from Baddiley et al.:[41] 200 mg (0.42 mmol, 1.0 equiv) of D-ribose-phosphate barium salt was dissolved in 5 mL of deionised water. The solution was cooled down in an ice bath and 50 mg (1.3 mmol, 3.0 equiv) of sodium borohydride was added slowly in batches. The reaction was allowed to reach room temperature before being stirred overnight. The mixture was cooled down again and quenched by the slow addition of 1 mL of glacial acetic acid. The pH of the solution was then adjusted to ~9 using dilute ammonia (1M).
5 g of AG1 X-8 resin (acetate form) was equilibrated with a solution of 0.1M ammonium acetate (pH 8) on a roller for 30 min. The supernatant was then removed and the sample applied and left incubating with the resin (on a roller) for 1h at room temperature. After removing the supernatant, the resin was washed twice with the ammonium acetate solution. 20 mL of 1N ammonium formate was then added to the resin and the mixture was incubated on a roller for 1h at room temperature to elute the product.
3 g of Amberlite IR-120 (H^+ form) was washed three times with 15 mL of 4% HCl and then three times with deionised water (until the pH of the supernatant was neutral). The eluate from the AG1 X-8 purification was then added to the Amberlite resin and incubated on a roller at room temperature for 1h, before being collected by filtration. The flow-through was lyophilised. To remove any traces of borate, the dry residue was redissolved in anhydrous methanol, stirred for 30 min, and the solvent was removed under reduced pressure. This process was repeated two more times. The dry residue was redissolved in deionised water, and 5 mL 0.1N barium hydroxide was added followed by a piece of dry ice. After the sample had set, the solid residue was removed by centrifugation. The supernatant was collected and an excess of ethanol was then used to precipitate RboP as its barium salt. The precipitate was washed with acetone and dried. **HRMS (ESI):** calculated for $C_5H_{12}O_8P$ $[M-H]^-$ 231.03, found 230.82. Characterisation was in accordance with the data reported in literature.[5,41]

Synthesis of 1-ethynyl-D-ribitol (Probe A, compound 16)

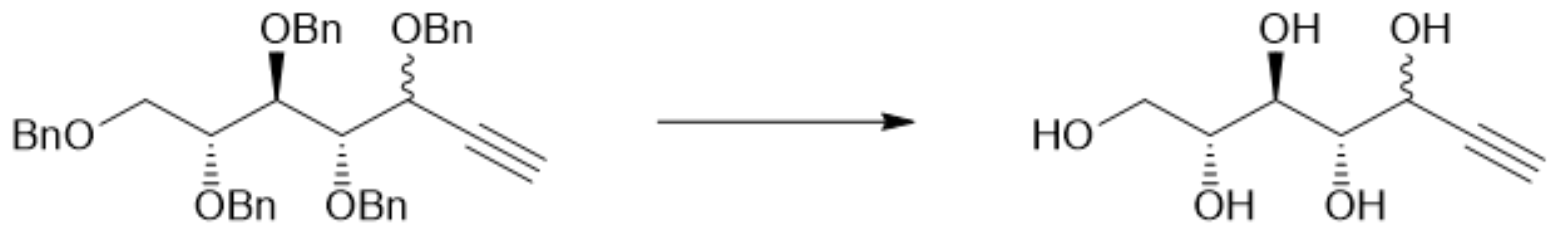

Fully benzylated 1-ethynyl-D-ribitol, described previously[22] (72 mg, 0.41 mmol, 1.0 equiv) was dissolved in anhydrous CH_2Cl_2 (3.8 mL) under N_2 atmosphere and cooled to –78 ℃. 1.0M BCl_3 in CH_2Cl_2 (3.8 mL, 3.8 mmol, 9.5 equiv) was added very slowly, and the mixture was stirred for 3 hours at –78℃. Upon completion, the reaction was quenched with 20:1 (v:v) MeOH/H_2O and stirred for 30 minutes before allowing it to warm to room temperature. The mixture was evaporated *in vacuo*. The sample was purified by reverse phase column chromatography using a C18 column on a Teledyne ACCQprep H150 prep HPLC system (10-80% H_2O/acetonitrile/0.1% formic acid) to obtain the product, Probe A, as a dark orange residue (23 mg, 0.13 mmol, 32%) in a 1.1:1 ratio of diastereoisomers. R_f 0.56 (1:4 MeOH/CH_2Cl_2). **HRMS (ESI)**: calculated for $C_7H_{12}NaO_5$ $[M+Na]^+$ 199.0582, found 199.0577. **^{1}H NMR** of isomeric mixture (400 MHz, DMSO-D_6) δ(ppm) 4.90 (d, J = 6.4 Hz, 1H, OH), 4.61 (d, J = 4.6 Hz, 1H, OH), 4.48 (t, J = 6.8 Hz, 2H, OH), 4.43 (dd, J = 5.0, 2.1 Hz, 1H), 4.35 (dq, J = 4.6, 2.3 Hz, 2H), 4.27 (dd, J = 8.0, 1.6 Hz, 1H, OH), 3.66 – 3.54 (m, 8H), 3.22 (dd, J = 8.7, 6.4 Hz, 1H, H7). **^{13}C NMR** (101 MHz, DMSO-D_6) δ(ppm) 107.6 (C6-major), 106.9 (C6-minor), 77.2 (C1-major), 77.0 (C1-minor), 75.2 (C4-major), 73.5 (C4-minor), 70.2 (C7), 69.8, 69.3, 65.5, 65.0, 63.6.

Synthesis of (2S,3S,4R)-2,3,4,5-tetrahydroxy-pent-1,6-yne (Probe B, compound 17)

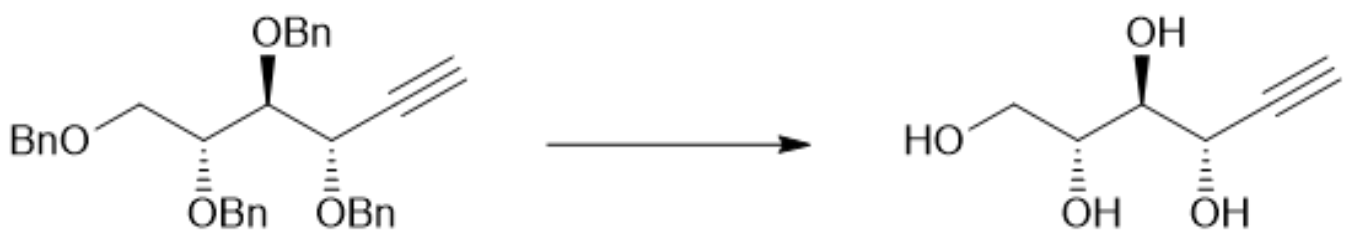

(2S,3S,4R)-2,3,4,5-tetrabenzyloxy-pent-1,6-yne, described previously[22] (0.15 g, 0.31 mmol, 1.0 equiv) was dissolved in anhydrous CH_2Cl_2 (10 mL) under N_2 atmosphere and cooled to –78 ℃. 1.0M BCl_3 in CH_2Cl_2 (3.1 mL, 3.1 mmol, 10 equiv) was added slowly, and the mixture was stirred at –78 ℃ for 1.5 hours. Upon completion, the reaction was quenched with 20:1 (v:v) MeOH/H_2O and stirred for 5 minutes before allowing it to warm to room temperature. The mixture was evaporated *in vacuo*. The sample was purified by silica column chromatography (0-20% MeOH in CH_2Cl_2) to obtain the product, Probe B (20 mg, 0.14 mmol, 47%). **HRMS (ESI)**: calculated for $C_6H_{10}KO_4$ $[M+K]^+$ 185.0216, found 185.0211. **^{1}H NMR** (400 MHz, CD_3OD) δ(ppm) 4.56 – 4.55 (t, *J* = 3.1 Hz, 1H, H2), 3.76 (dd, *J* = 11.1, 2.7 Hz, 1H, H5), 3.68 – 3.56 (m, 3H, H3 + H4 + H5), 2.77 (d, *J* = 2.2 Hz, 1H, H6). **^{13}C NMR** (101 MHz, CD_3OD) δ(ppm) 82.9 (C6), 75.4 (C4), 75.4 (C2), 73.6 (C3), 64.9 (C2), 64.5 (C5).

Expression and purification of recombinant enzymes

The sequence encoding TARI1_STAA8 from *S. aureus* was optimised for *E. coli* expression and ordered from Genscript.

Forward primer 5'-CCGCGCGGCAGCCATATGAAGTATGCGGGTATCCTGGC-3' and reverse primer 5'-AGTCATGCTAGCTTAGTCGTCCGCAATACCACCA-3' were used to clone the TarI gene into a pET-28a(+) vector using In-Fusion Cloning kit (TakaraBio). The ligation product was confirmed using Next-Gen Whole Plasmid Sequencing (Eurofins).

The sequences encoding human FGGY and PCYT2 were optimised for bacterial expression and ordered from Genscript in pET-28a(+).

Expression conditions used for all enzymes were identical: 5 ng of the plasmid was used to transform 50 µL of BL21(DE3)-Gold chemically competent cells (New England Biolabs) by heat shock following standard protocols. Cultures were plated on LB-agar plates with kanamycin (50 µg/mL) and selected at 37 °C for 16 h. Starter cultures were prepared by picking single colonies into 100 mL of LB with kanamycin (50 µg/mL) and grown at 37 °C for 16 h with shaking (180 rpm). Flasks containing LB with 50 µg/mL kanamycin (3 x 1L of media) were inoculated with the starter culture at a dilution of 1:100 (10 mL per 1 L of media) and grown at 37 °C with shaking (180 rpm) until OD600 reached ~0.6. At that point the cultures were induced with isopropyl β-D-1-thiogalactopyranoside (IPTG) to a final concentration of 1 mM and maintained shaking (180 rpm) at 18 °C overnight.

For the purification of the enzymes, the cells were harvested by centrifugation (6,000 g, 4 °C, 10 min) and the pellets were resuspended in 200 mL lysis buffer (25 mM Tris HCl, 300 mM NaCl, 5 mM imidazole, pH 7.8 with protease inhibitor cocktail). The cells were lysed by sonication (30 sec on, 30 sec off), centrifuged (27,000 g, 4 °C, 40 min), and the enzymes were purified from their respective lysates by immobilised metal affinity chromatography using a HisTrap FF Crude 5 mL column (Cytiva) and a gradient of elution buffer (25 mM Tris HCl, NaCl 300 mM, imidazole 500 mM). The eluted fractions were analysed by SDS-PAGE and the fractions containing the target enzyme were pooled and desalted on a HiPrep G-25 desalting column (Cytiva) into storage buffer (25 mM Tris HCl, NaCl 150 mM, pH 7.8). Relevant protein fractions were pooled and concentrated using 10 kDa MWCO centrifugal filters. Typical protein yields were 40 mg of FGGY and 7 mg of TarI or PCYT2 per 1 L of expression culture. The enzymes were stable in storage buffer and could be aliquoted and stored in the -40 °C freezer, except for His-PCYT2, which required the addition of 20% glycerol to stabilise it. Since glycerol would interfere with the downstream applications of PCYT2, before each enzymatic reaction the thawed aliquots of the enzyme were desalted on a 5mL HiTrap G-25 desalting column (Cytiva) into a glycerol-free buffer and used immediately.

Enzymatic synthesis of CDP-ribitol

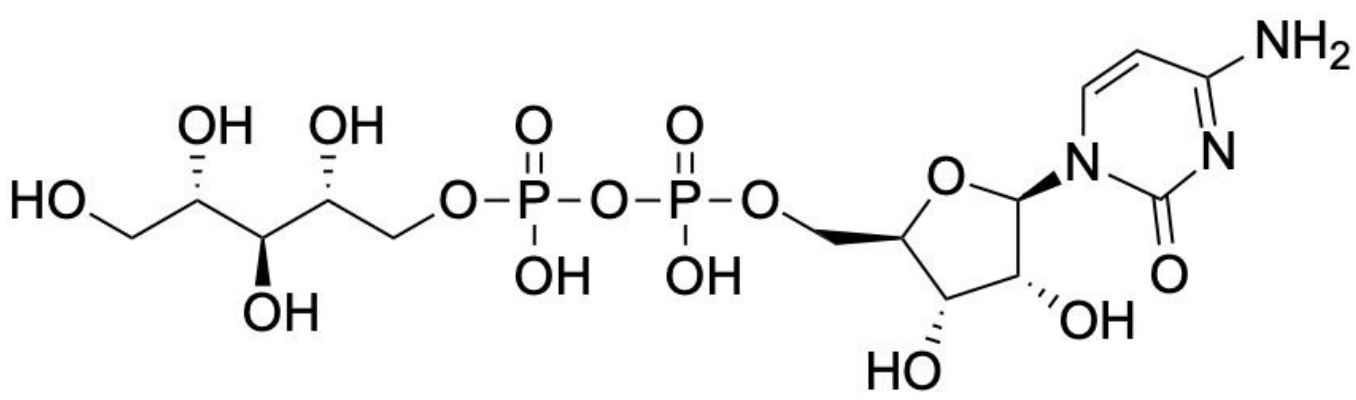

Note: ATP and CTP were prepared as stock solutions at a concentration of 500 mM buffered with Tris base to a pH between 7.3 and 7.5. We found this step was important since using unbuffered stock solutions resulted in acidification of the reaction mixture and caused precipitation of the proteins.

Seven identical reactions were set up in Eppendorf tubes, each containing ribitol at a final concentration of 10 mM (0.010 mmol per reaction), ATP, CTP, and $MgCl_2$ at a final concentration of 25 mM each, and His-FGGY and His-TarI at a final concentration of 5 μM each in a total volume of 1 mL of reaction buffer (Tris HCl 25 mM, NaCl 150 mM, pH 7.4). The tubes were incubated in a thermal shaker at 37 ℃/600 rpm overnight. The reaction was stopped by boiling the samples at 95 ℃ for 10 min. The samples were then spun down and the pellet discarded. To each sample was added 25 units of FastAP alkaline phosphatase (ALP), along the supplier-provided reaction buffer, and the samples were incubated in a thermal shaker at 37 ℃/600 rpm overnight. The reaction was stopped by boiling the samples at 95 ℃ for 10 min then the samples were spun down and the pellet discarded. Small aliquots were used to confirm the disappearance of the nucleotides from the reaction mixture by LC-MS, before the samples were pooled and run through a 3 kDa MWCO spin concentrator to remove any remaining protein. The flow-through was collected and its pH adjusted to ~9 with dilute ammonia (1M). The sample was then added to 3 g of AG-1 x8 resin (acetate form) and gently agitated at room temperature for 1h. The supernatant was discarded and the resin was washed twice with deionised water. The product was eluted with 20 mL of 1N ammonium formate, after which the eluate was lyophilised.

The dry residue was weighed to determine the loading for preparative HPLC purification. The sample was then dissolved in an appropriate volume of HPLC-grade water containing 0.1% formic acid and was purified on a Teledyne ACCQprep H150 prep HPLC system using a C18AQ RediSep column (30x150 m, 5 μm) as a stationary phase and water/acetonitrile (each containing 0.1% FA) as a mobile phase. The run was set at an isocratic step of 100% water (0.1% FA) at a flow rate 42.5 mL/min for 3 column volumes (CVs), followed by a stepped gradient of acetonitrile (0.1% FA) for 4 CVs, and finally an isocratic step of 100% acetonitrile (0.1% FA) for 2 CVs. Peak fractions were analysed by LC-MS, and the target compound was found to have eluted in the first isocratic step of 100% water. The fractions containing the product were pooled and lyophilised to give CDP-ribitol in a yield of 16.6 mg (0.031 mmol, 44%). **ESI-MS:** calculated for $C_{14}H_{24}N_3O_{15}P_2$ $[M-H]^-$ 536.08, found 536.04. **^{1}H NMR** (400 MHz, D_2O): δ(ppm) 8.11 (d, *J* = 7.9 Hz, 1H), 6.23 (d, *J* = 7.8 Hz, 1H), 5.92 (d, *J* = 3.2 Hz, 1H), 4.23-4.235 (m, 4H), 3.89-4.21 (m, 3H), 3.68–3.93 (m, 4H), 3.65-3.65 (m, 1H). **^{13}C NMR** (101 MHz, D_2O) δ(ppm) 169.81, 161.22, 143.36, 95.69, 89.49, 83.11 (d, *J* = 8.7 Hz), 74.35, 72.08, 71.62, 70.86 (d, *J* = 7.8 Hz), 69.16, 67.01 (d, *J* = 5.8 Hz), 64.47, 62.33. **^{31}P NMR** (162 MHz, D_2O) δ 10.27 (d, *J* = 112 Hz).

General method for the enzymatic synthesis of CDP-derivatives

ATP and CTP were prepared as stock solutions at a concentration of 500 mM buffered with Tris base to a pH between 7.3 and 7.5.

To a reaction mixture (final volume 100 μL) containing ATP, CTP and $MgCl_2$ each at a final concentration of 25 mM and the kinase and cytidylytransferase both at a final concentration of 5 μM in reaction buffer (Tris HCl 25 mM, NaCl 150 mM, pH 7.4), substrate was added at a final concentration of 10 mM, unless indicated otherwise. The reaction tube was incubated in a thermal shaker at 37 ℃/600 rpm overnight. The reaction was then stopped by boiling the sample at 95 ℃ for 10 min. The sample was then spun down and the pellet discarded. The supernatant was used to analyse product formation by LC-MS as described below. Quantitative analysis could not be performed due to the lack of pure products to use as standards. Instead, relative peak intensities in the extracted ion chromatograms were used to qualitatively assess the level of conversion.

Individual FGGY and TarI reactions were performed essentially as above, but using ribitol as a substrate with FGGY and ATP (without TarI/CTP), or synthetic RboP as a substrate with TarI and CTP (without FGGY/ATP). Control reactions were performed without enzyme or with enzyme that had been denatured by boiling at 95 ℃ for 10 min.

LC-MS analysis of the enzymatic reaction products

High Performance Liquid Chromatography Electrospray Ionisation Mass Spectrometry (HPLC-MS) analysis of the CDP derivatives was performed using a Dionex UltiMate® 3000 Ci Rapid Separation LC system equipped with an UltiMate® 3000 photodiode array detector probing at 250–400 nm, coupled to a HCT ultra ETD II (Bruker Daltonics) ion trap spectrometer, using Chromeleon® 6.80 SR12 software (ThermoScientific), esquireControl version 6.2, Build 62.24 software (Bruker Daltonics), and Bruker compass HyStar 3.2-SR2, HyStar version 3.2, Build 44 software (Bruker Daltonics). All mass spectrometry was conducted in negative ion mode. Data analysis was performed using ESI Compass 1.3 DataAnalysis, Version 4.1 software (Bruker Daltonics). Samples were chromatographically analysed using an Accucore HILIC column, 2.6 µ (50 mm × 2.1 mm, Thermoscientific). Samples from the reaction mixtures were run crude without any further treatment. Water, 0.1% formic acid by volume (solvent A), and acetonitrile, 0.1 % formic acid (solvent B) were used as the mobile phase at a flow rate of 0.3 $mL.min^{-1}$ at 30 °C. A 1-minute step with 95% solvent B was used to load the sample onto the column, then a gradient from 95% to 5% solvent B over 5 minutes was used to elute the target compound. The mobile phase was held at 5% solvent B for 2.5 minutes before shifting it back to 95% and equilibrating the column again at that concentration for 2 minutes for the next run.

LC-MS/MS quantitative analysis of CDP-ribitol

High Performance Liquid Chromatography-Tandem Mass Spectrometry (HPLC-MS/MS) was performed on a Dionex UltiMate 3000 UHPLC system (ThermoScientific) coupled to a TSQ Endura triple quadrupole mass spectrometer (ThermoScientific) via a heated electrospray ionisation (H-ESI) source operated in negative ion mode. For the standard calibration curve, a series of standard solutions with known concentrations of pure CDP-ribitol were prepared in LC-MS grade water then analysed. For analysing samples from the stability study, an aliquot was taken from the incubated solution, diluted with LC-MS grade water at a ratio of 1:1, then analysed. Sample analysis was performed using an Accucore HILIC column, 2.6 µ (50 mm × 2.1 mm, Thermoscientific). Samples from the reaction mixtures were run crude without any further treatment. Water, 0.1% formic acid by volume (solvent A), and acetonitrile, 0.1 % formic acid (solvent B) were used as the mobile phase at a flow rate of 0.3 $mL.min^{-1}$ at 30 °C. A 1-minute step with 95% solvent B was used to load the sample onto the column, then a gradient from 95% to 5% solvent B over 5 minutes was used to elute the target compound. The mobile phase was held at 5% solvent B for 2.5 minutes before shifting it back to 95% and equilibrating the column again at that concentration for 2 minutes for the next run. Mass spectrometric detection was performed using the following source parameters: spray voltage, 2500 V; sheath gas, 50 (arbitrary units); auxiliary gas, 10; sweep gas, 1; ion transfer tube temperature, 325 °C; and vaporiser temperature, 350 °C. CDP-ribitol was quantified by selected reaction monitoring (SRM) of the transition m/z 536.038 to 321.982, with a collision energy of 20.33 V and an RF lens voltage of 184 V, acquired between 0.5 and 12.0 min. Q1 and Q3 resolutions were set to 0.7 and 1.2 Da (FWHM), respectively. Data were acquired and processed using Xcalibur version 4.0.27.10 (ThermoScientific).

Supporting figures

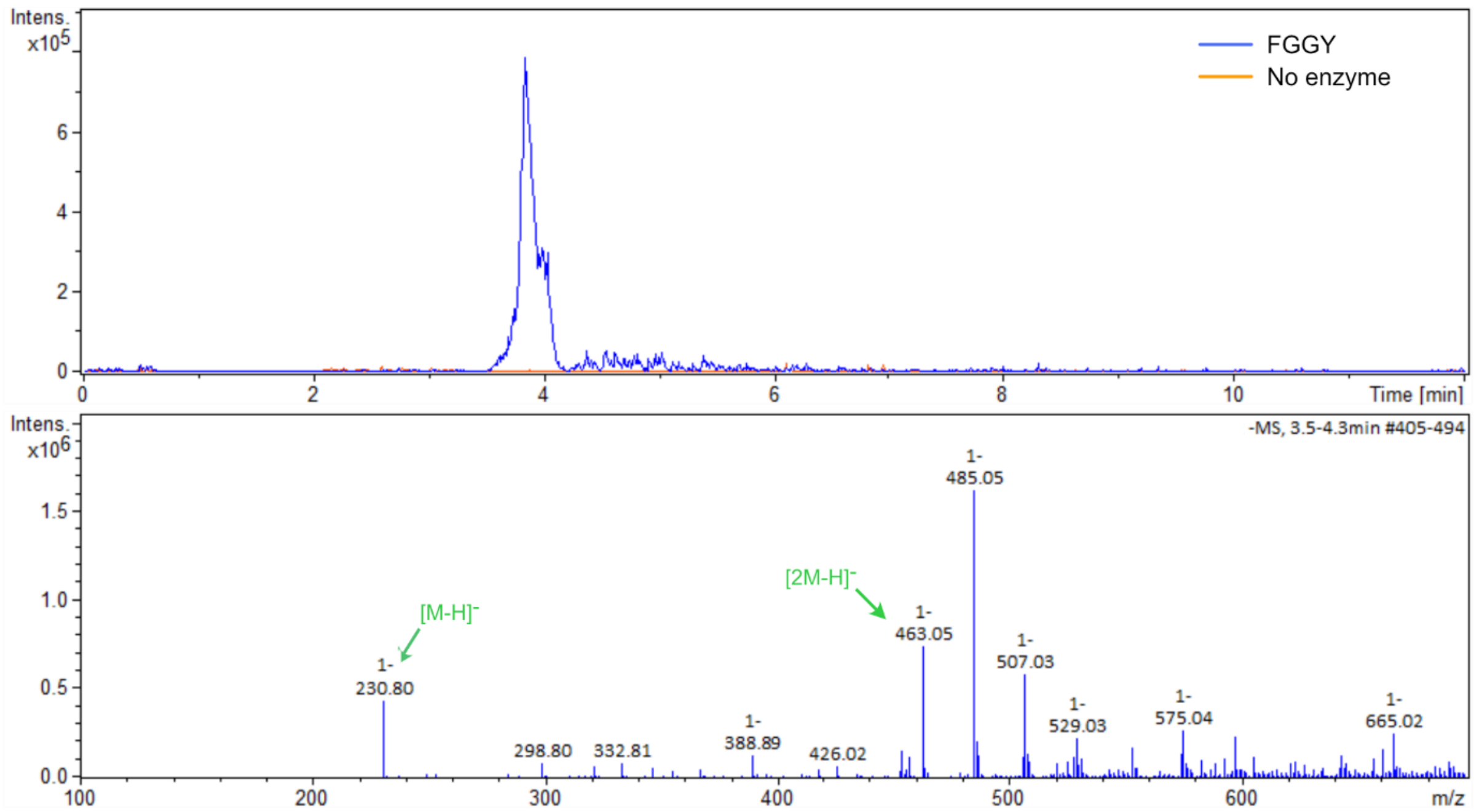

Figure S1. Top: extracted ion chromatogram (EIC) of RboP from a sample of ribitol treated with FGGY and an untreated (no enzyme) control. Bottom: mass spectrum of the FGGY-treated sample, showing the formation of RboP (m/z 463.05, calculated 463.06 ([2M-H]$^-$)).

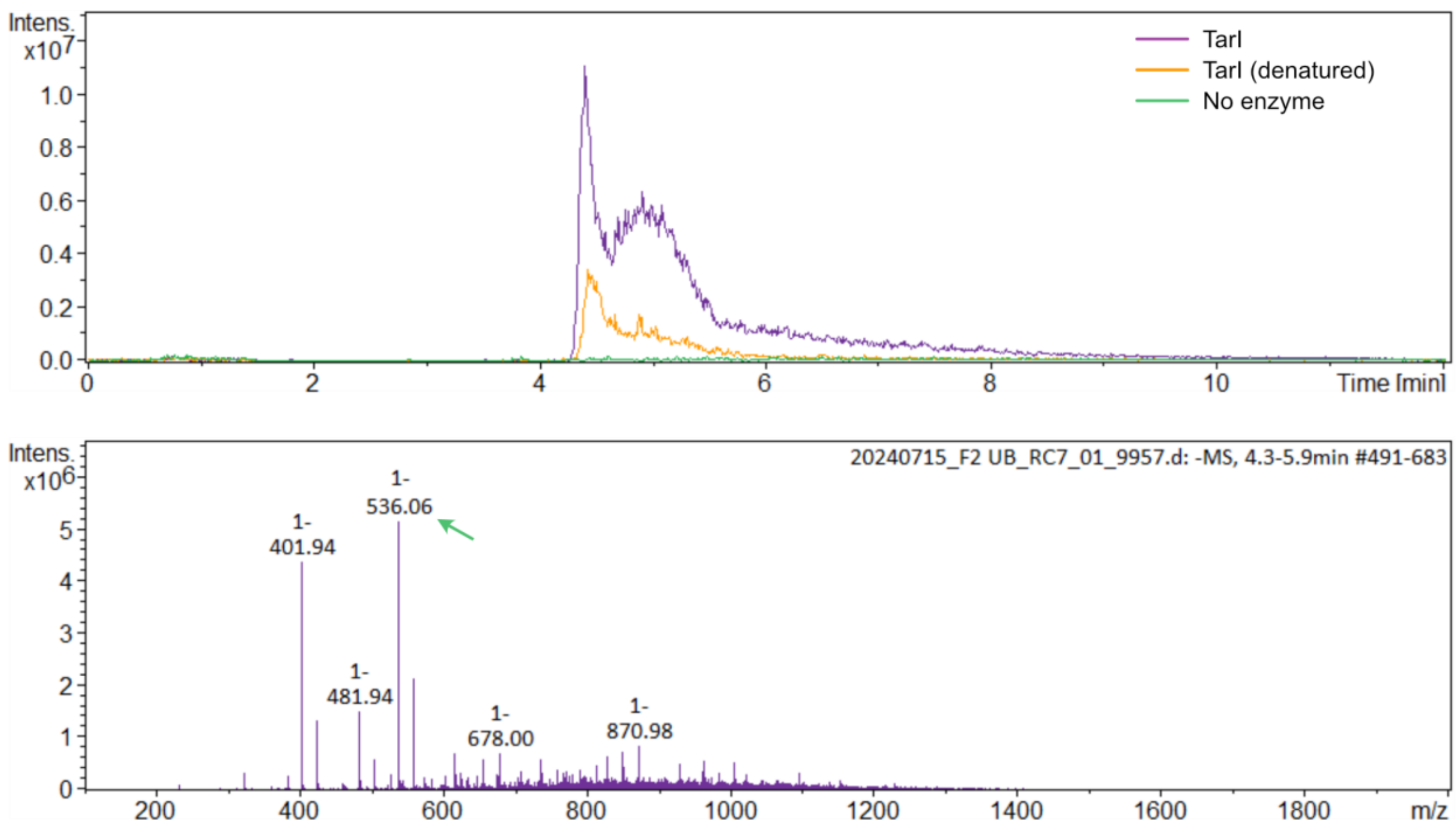

Figure S2. Top: EIC of CDP-ribitol from a sample of chemically synthesised RboP treated with TarI, an untreated (no enzyme) control and a control treated with denatured enzyme. Bottom: mass spectrum from the sample treated with active enzyme, showing the formation of CDP-ribitol (m/z 536.06, calculated 536.07 ([M-H]$^-$)).

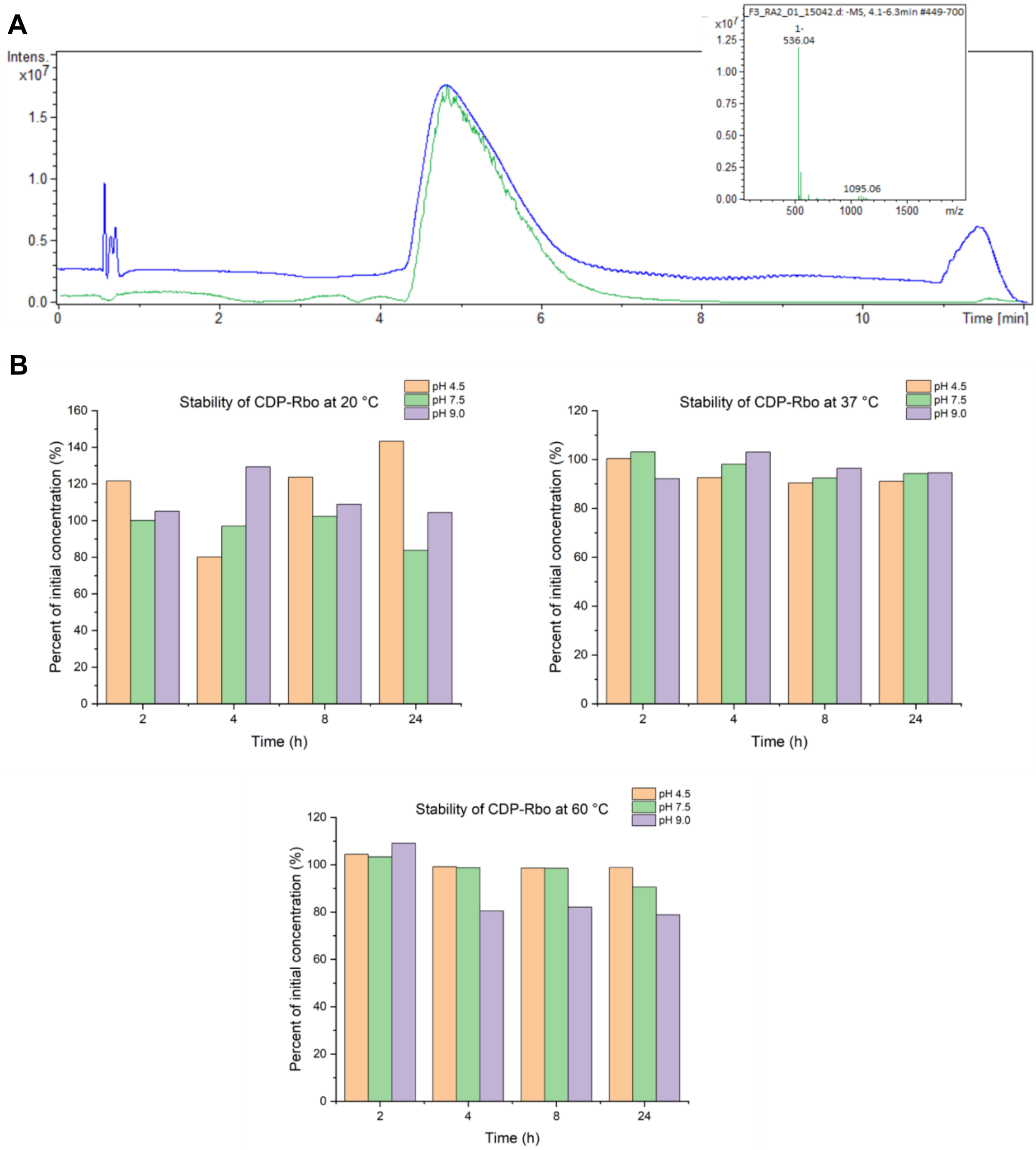

Figure S3. A) LC-MS chromatogram (green) and 280 nm UV trace (blue) of purified CDP-ribitol. The inset shows the mass spectrum corresponding to the peak, showing the expected mass of CDP-ribitol (m/z 536.04, calculated 536.07 ([M-H]$^-$)). B) Stability of CDP-ribitol during overnight incubation in buffers of different pH values and at different temperatures. Measurements were done using LC-MS/MS and CDP-ribitol levels were quantified using a standard curve prepared from purified CDP-ribitol. Values were normalised to the concentration of CDP-ribitol at the start of the experiment.

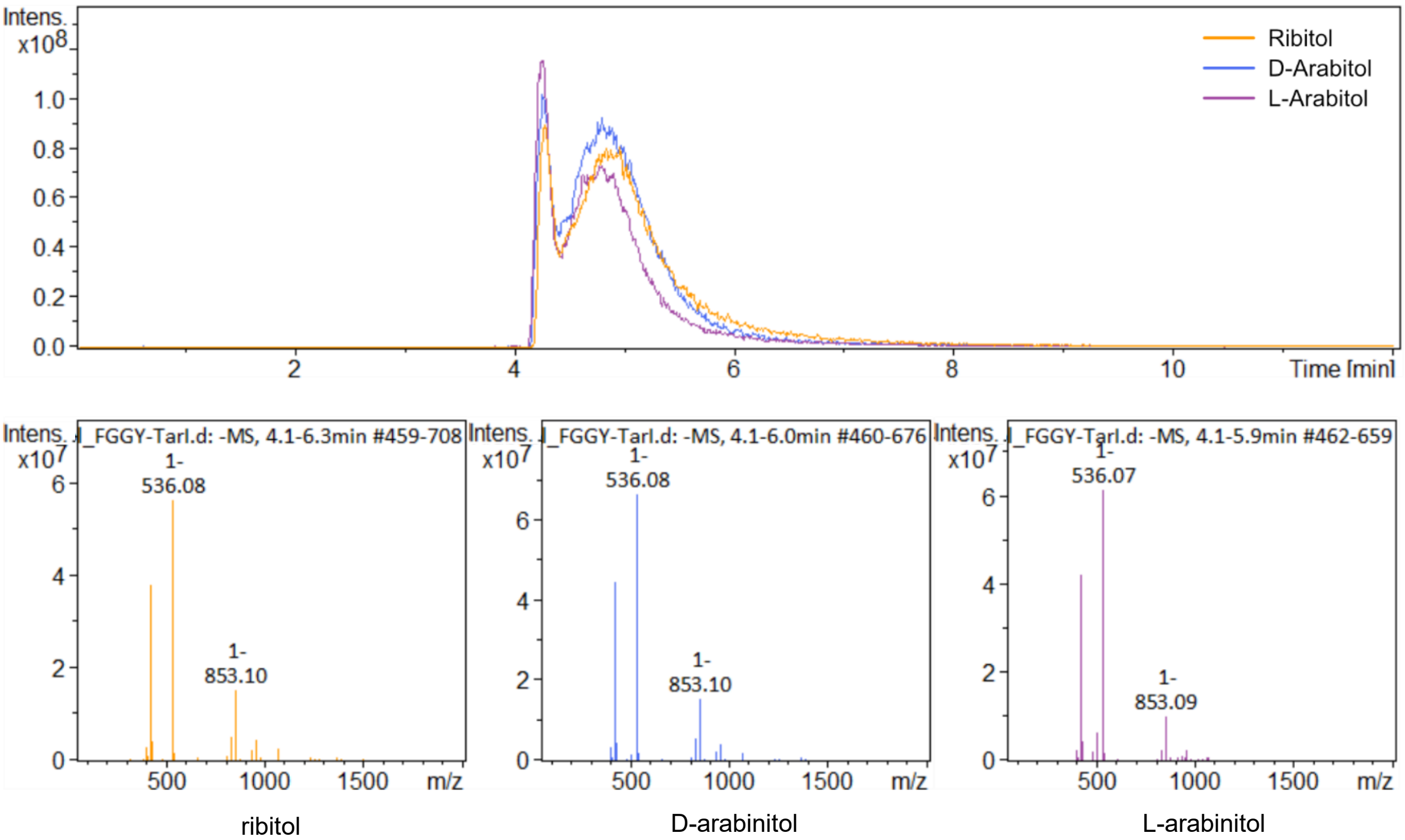

Figure S4. Top: EICs of CDP-conjugates of ribitol, D-arabinitol, and L-arabinitol obtained by reaction with FGGY/TarI. Bottom: corresponding mass spectra, showing the expected mass of each CDP conjugate (m/z 536.07/536.08, calculated 536.07 ($[M-H]^-$)).

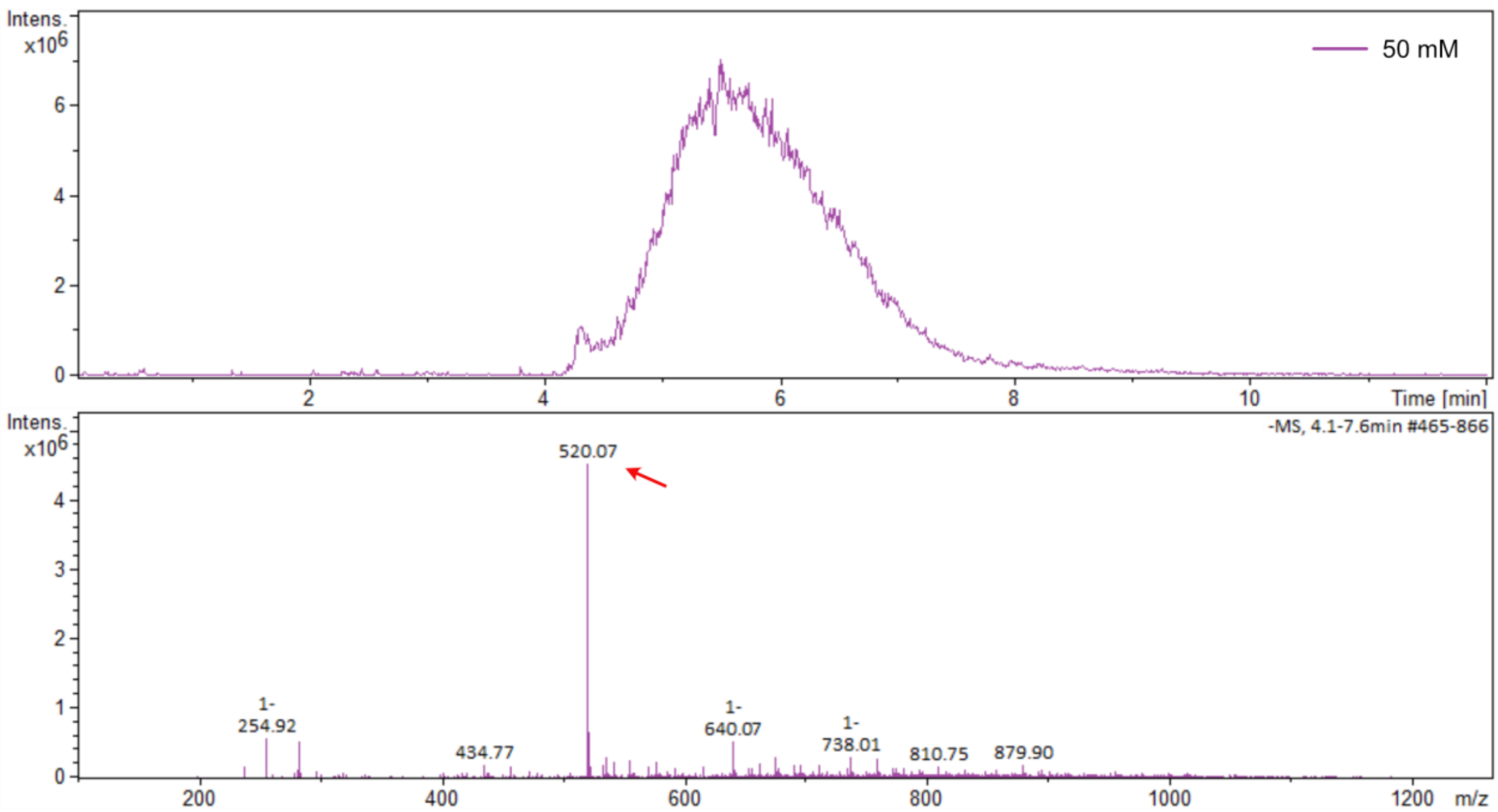

Figure S5. Top: EIC of the CDP-conjugate of methylerythritol obtained by reaction with FGGY/TarI. The sample was treated with ALP to reduce the background noise from the free nucleotides. Bottom: corresponding mass spectrum, showing the expected mass of the product (m/z 520.07, calculated 520.30 ($[M-H]^-$)).

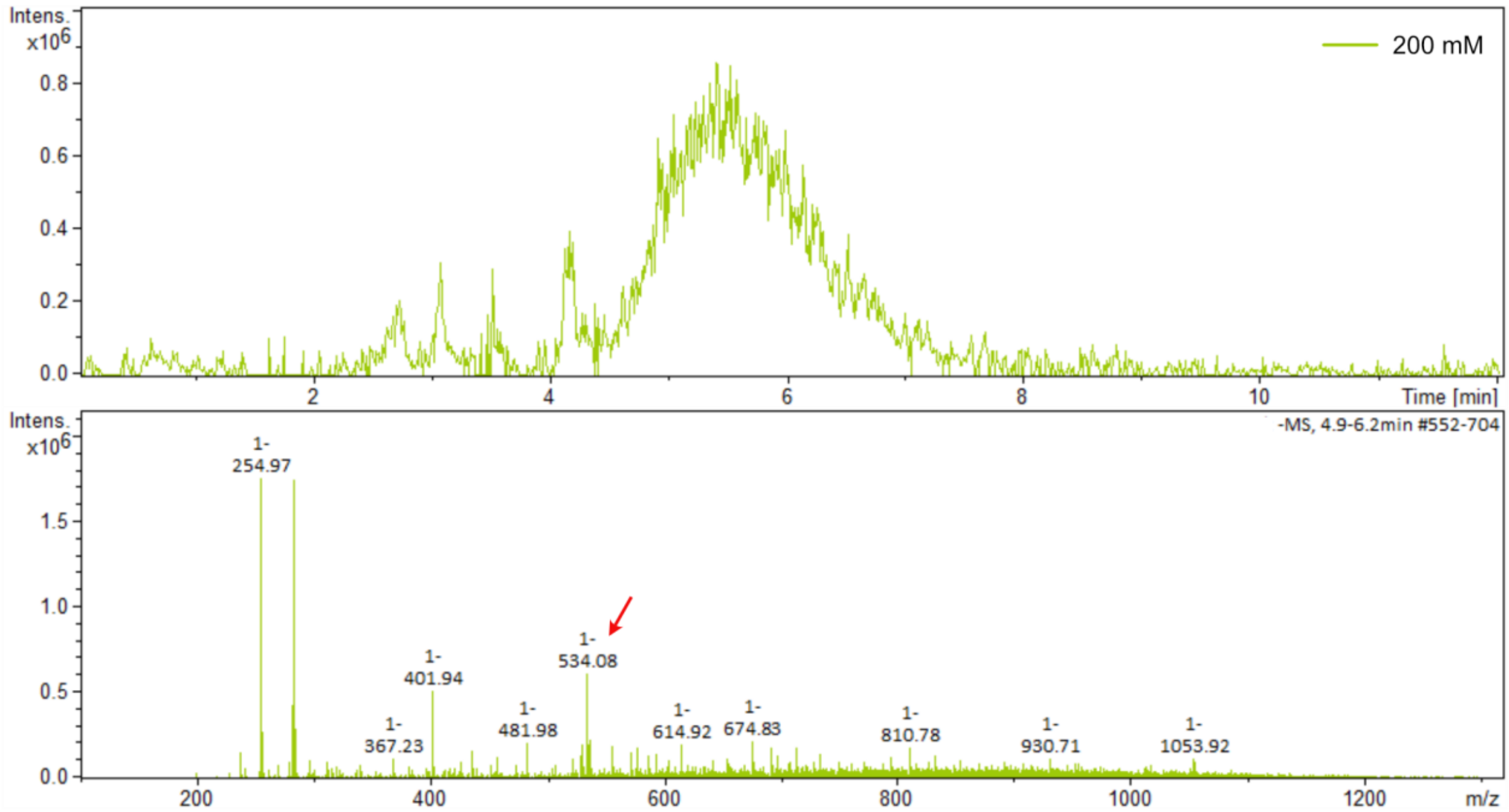

Figure S6. Top: EIC of the CDP-conjugate of D-xylulose obtained by reaction with FGGY/TarI. The sample was treated with ALP to reduce the background noise from the free nucleotides. Bottom: corresponding mass spectrum, showing the expected mass of the product (m/z 534.08, calculated 534.05 ($[M-H]^-$)).

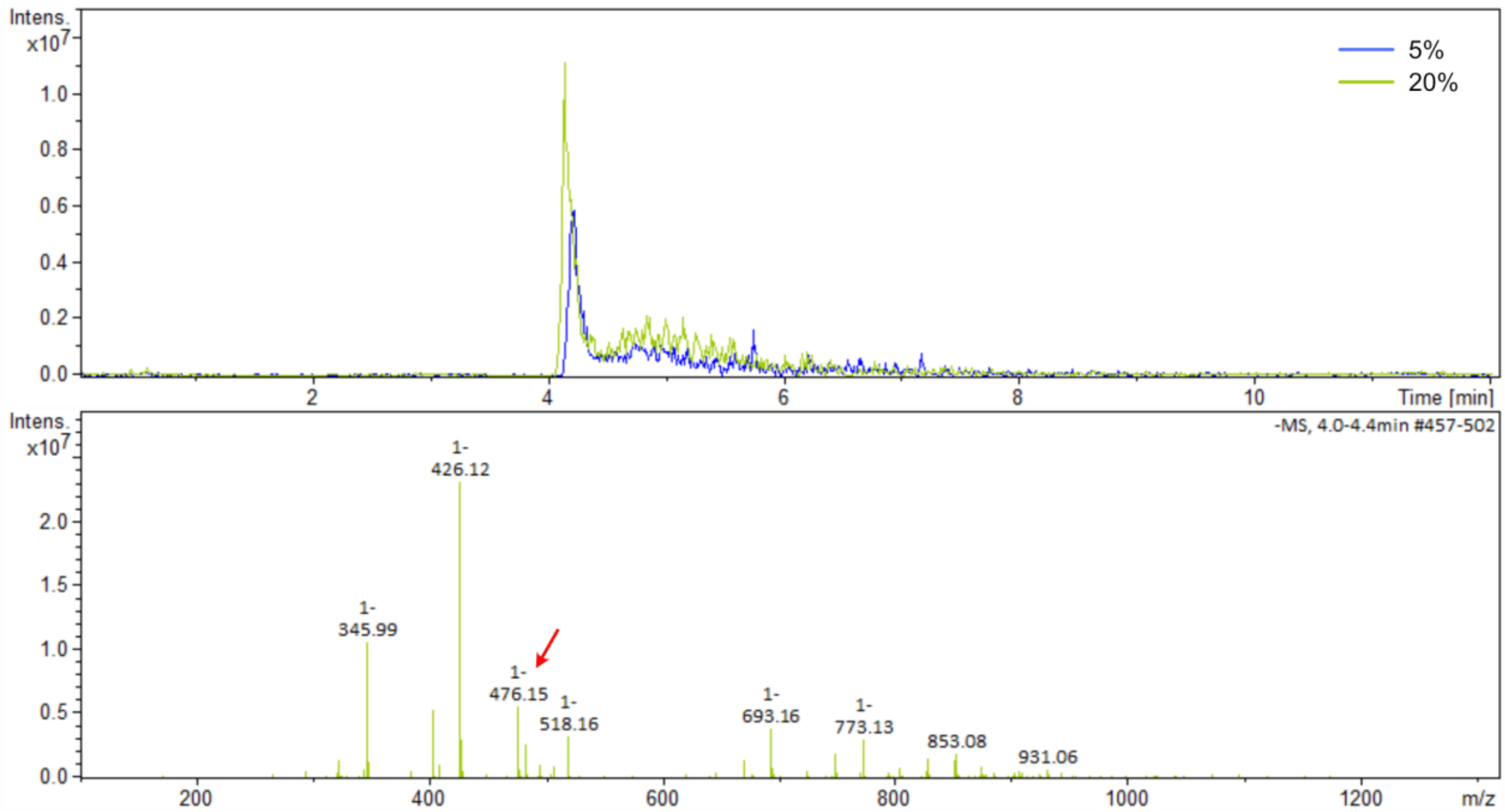

Figure S7. Top: EICs of CDP-glycerol obtained by reaction of FGGY/PCYT2 with different concentrations of glycerol. Bottom: mass spectrum corresponding to the sample with the highest concentration of the substrate, showing the conversion to its CDP-derivative (m/z 476.15, calculated 476.05 ($[M-H]^-$)).

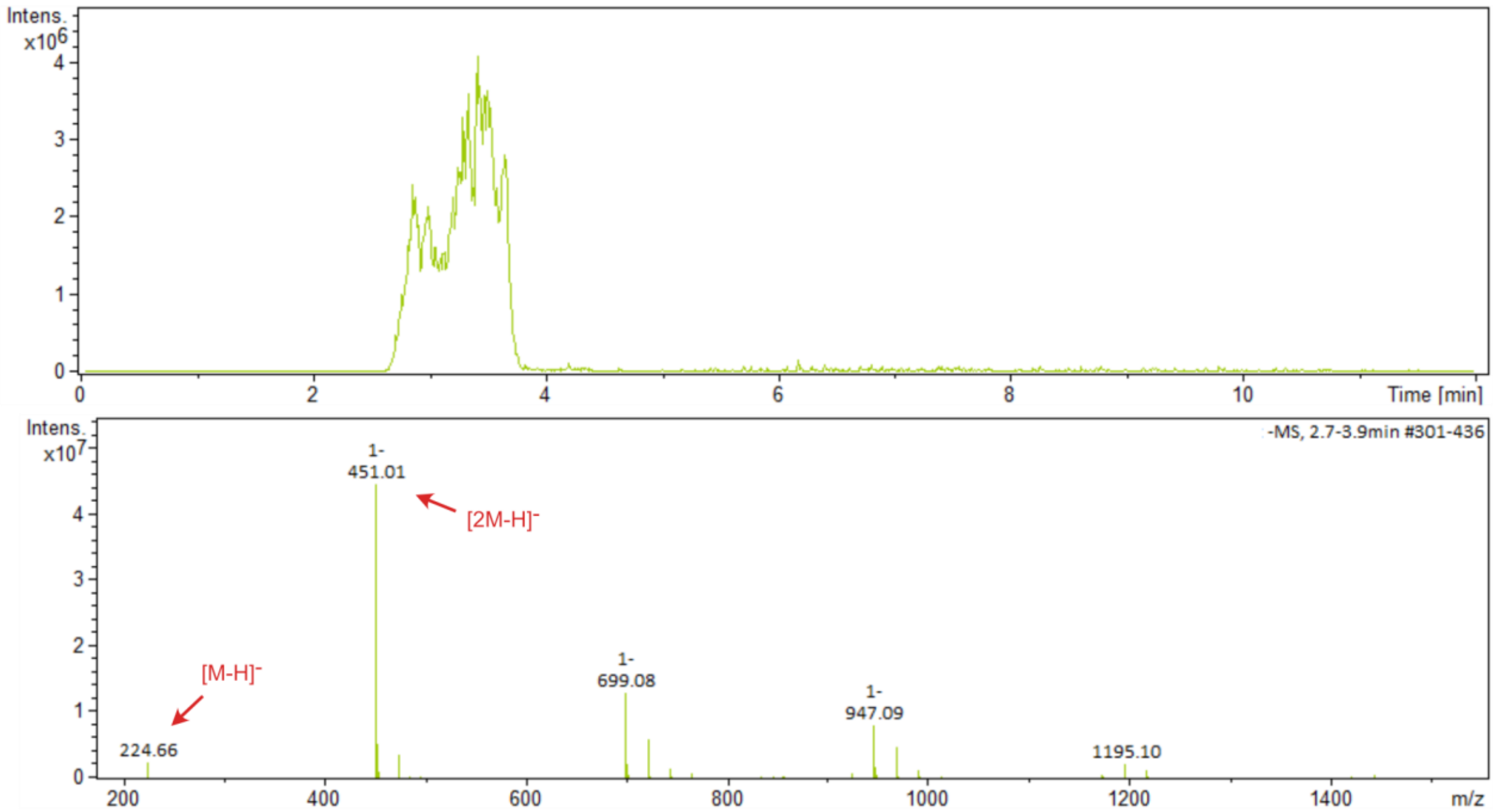

Figure S8. Top: EIC of the phosphate derivative of probe B from a reaction with FGGY/TarI. Bottom: corresponding mass spectrum, showing the formation of the phosphorylated product (m/z 451.01, calculated 451.04 ([2M-H]$^-$)).

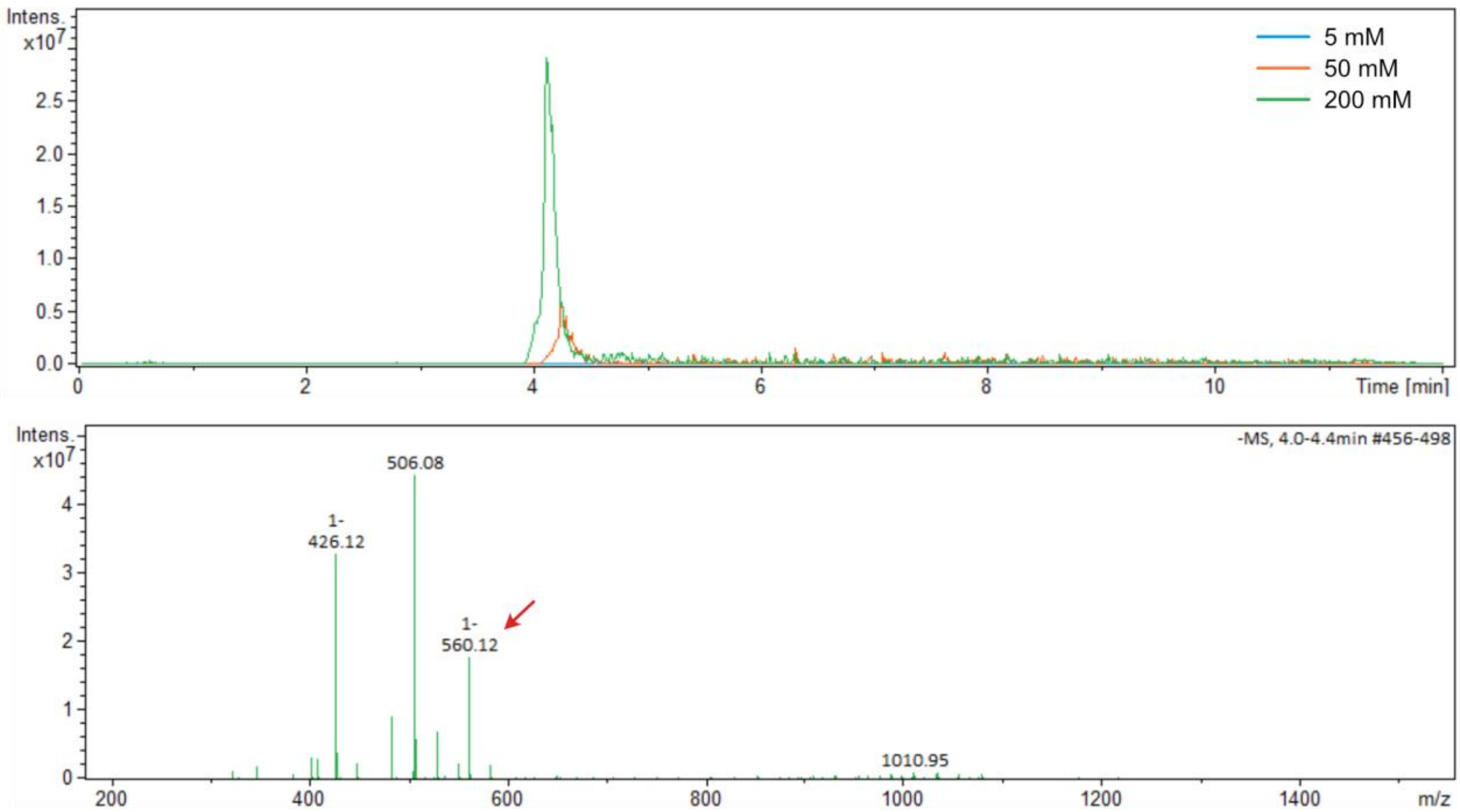

Figure S9. Top: EIC of the CDP-conjugate of probe A obtained from the reaction of FGGY/TarI with different concentrations of probe A. Bottom: mass spectrum corresponding to the sample with the highest concentration of the substrate, showing the conversion to its CDP-derivative (m/z 560.12, calculated 560.07 ([M-H]$^-$)).

NMR spectra

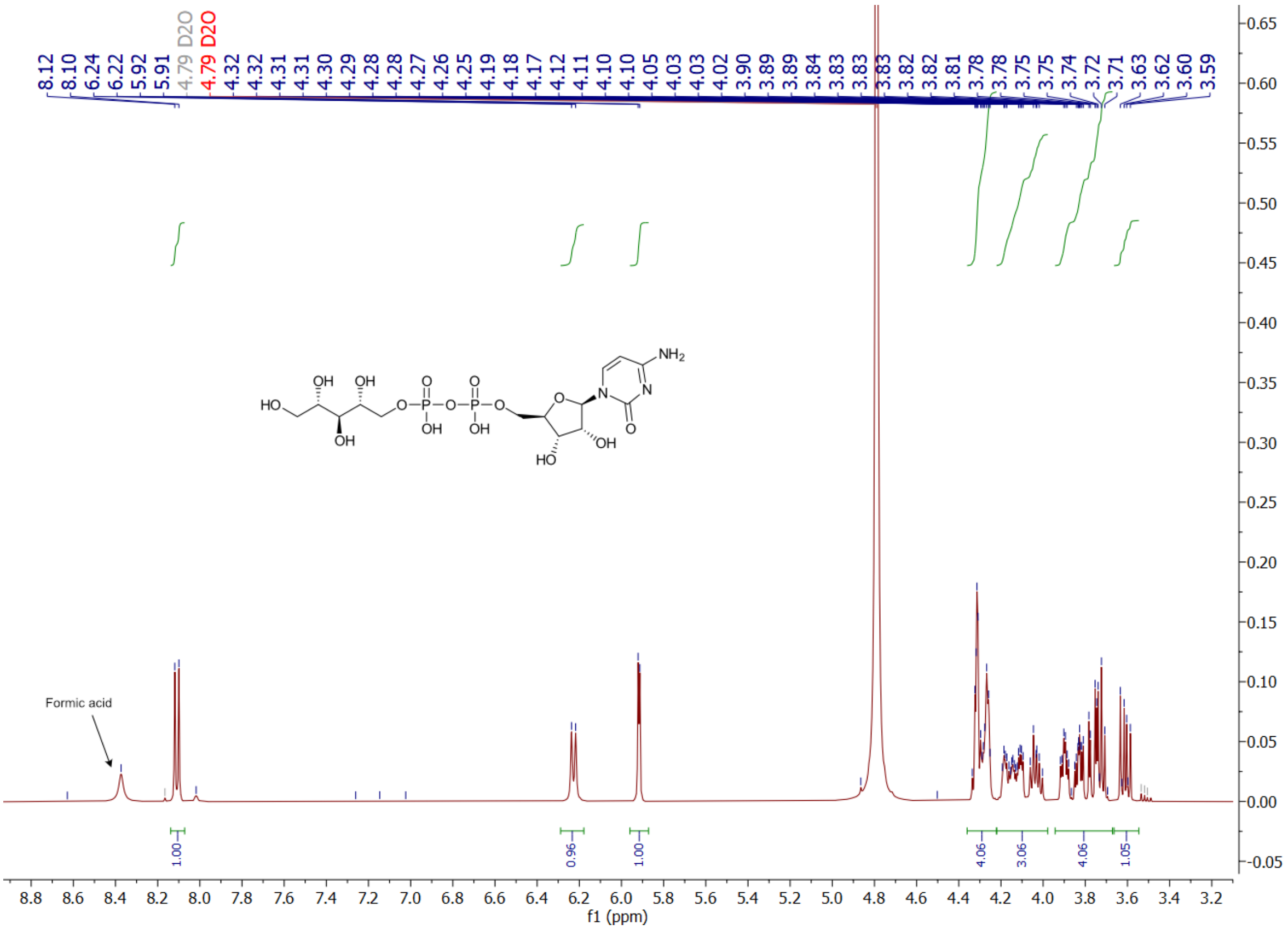

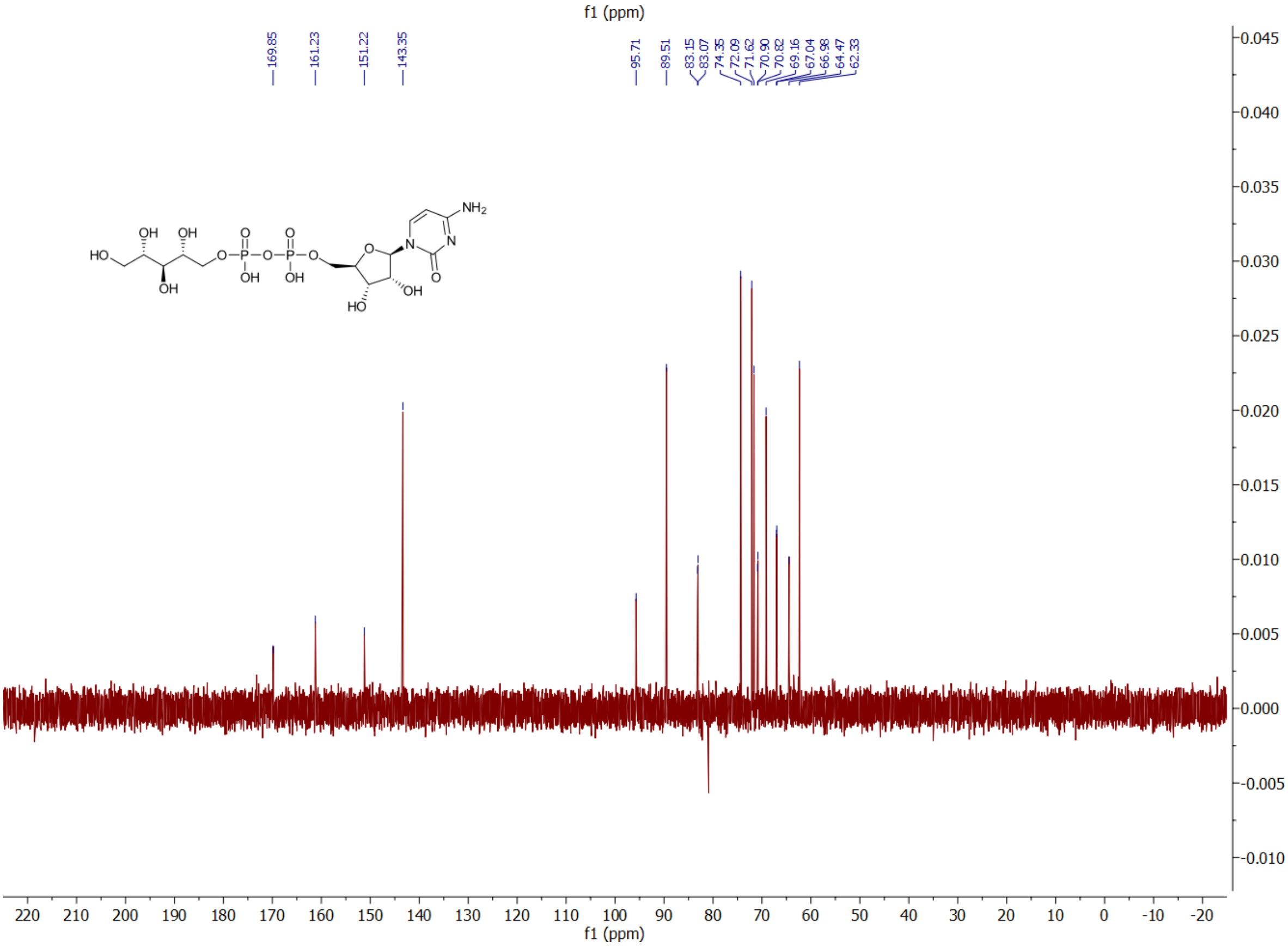

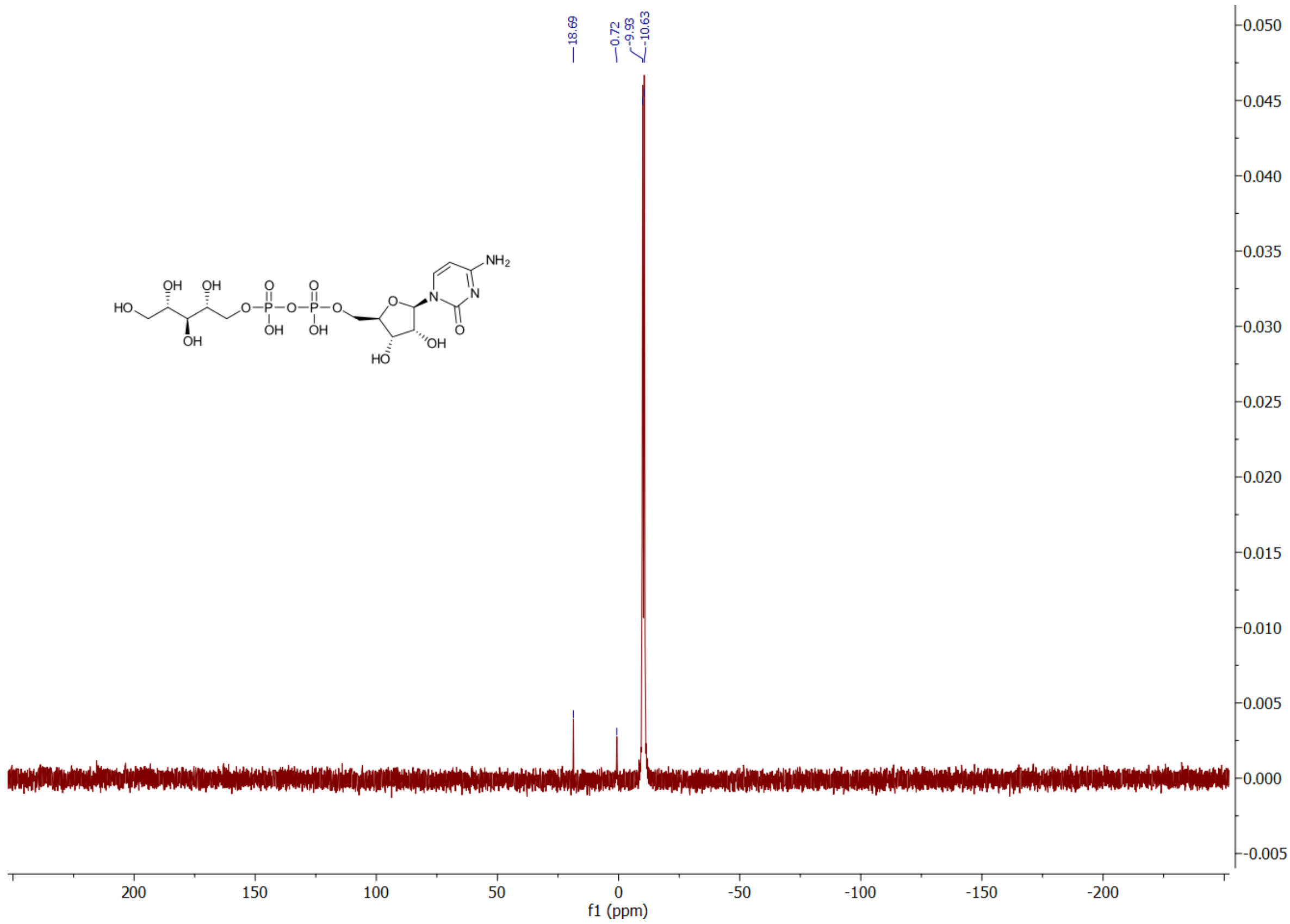
18.69
0.72
-9.93
-10.63
NH2
N
O
HO
OH
P
f1 (ppm)

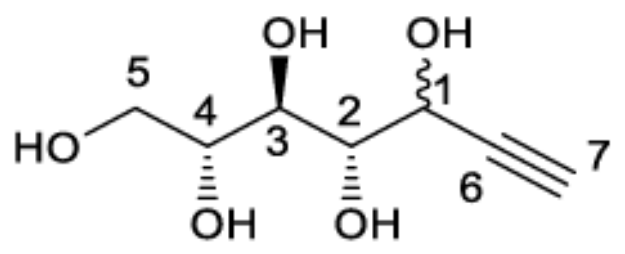
OH
OH
5
4
3
2
1
HO
7
6
OH
OH

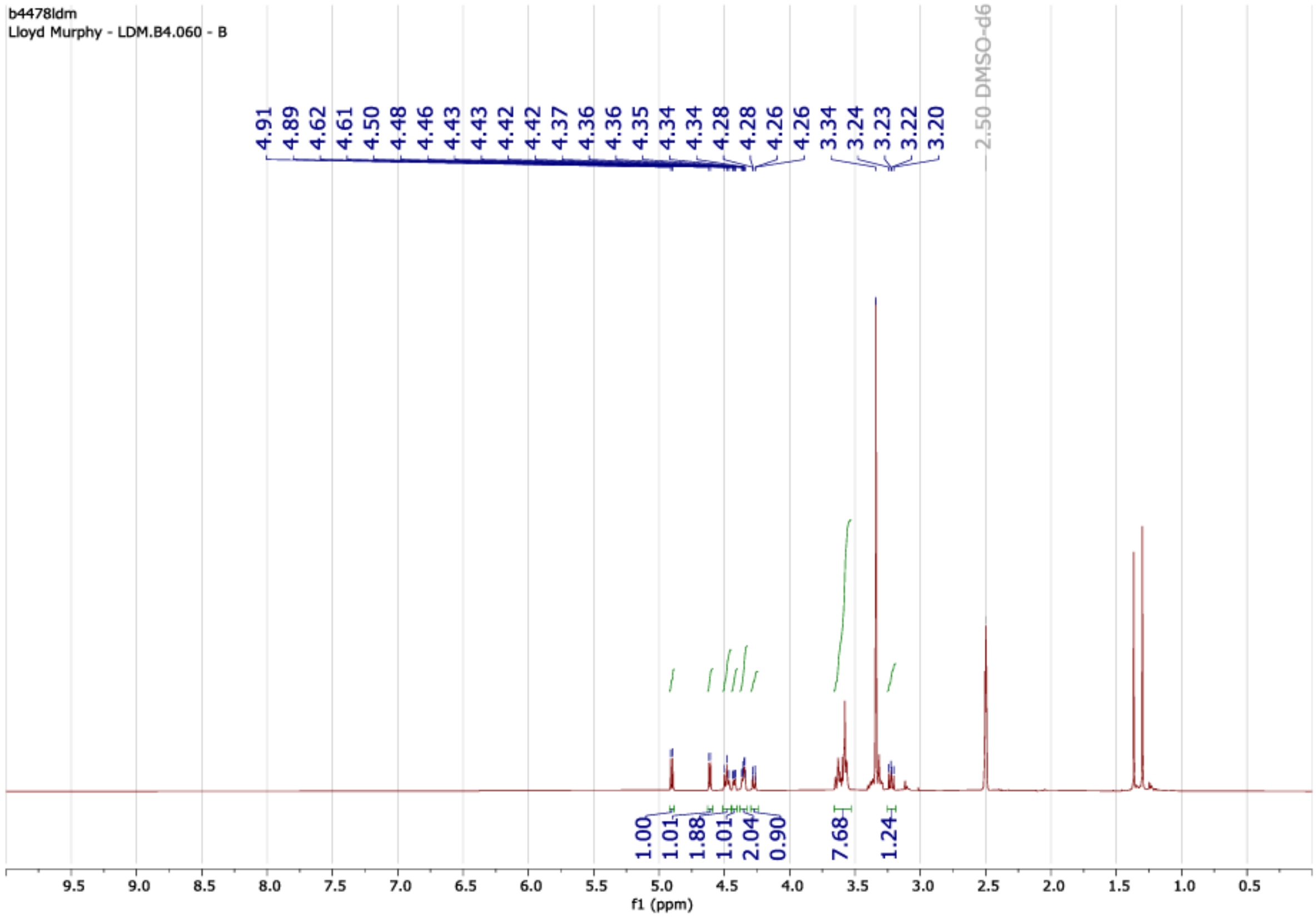
b4478ldm
Lloyd Murphy - LDM.B4.060 - B
4.91
4.89
4.62
4.61
4.50
4.48
4.46
4.43
4.43
4.42
4.42
4.37
4.36
4.36
4.35
4.34
4.34
4.28
4.28
4.26
4.26
3.34
3.24
3.23
3.22
3.20
2.50 DMSO-d6
1.00
1.01
1.88
1.01
2.04
0.90
7.68
1.24
f1 (ppm)

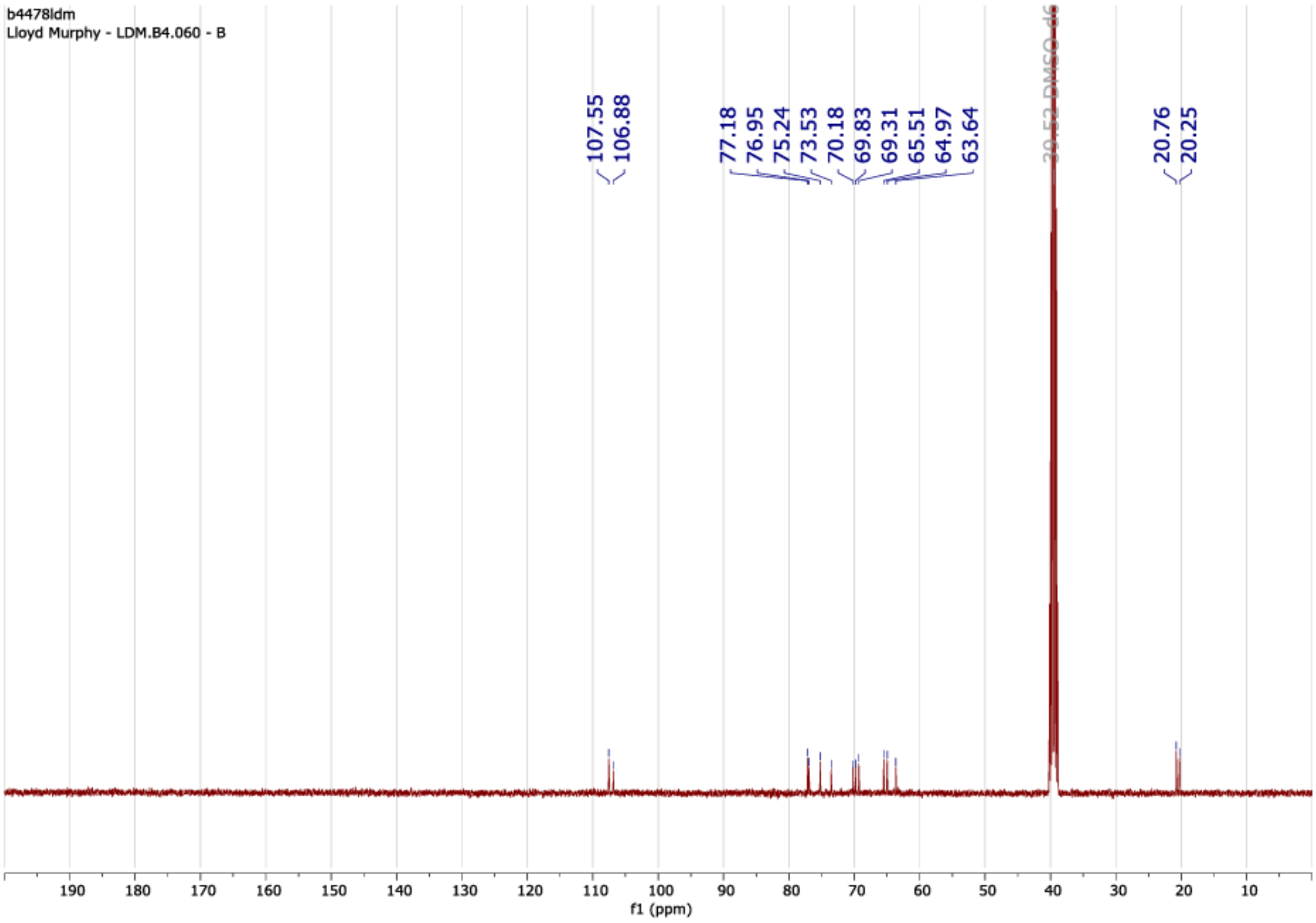
b4478ldm
Lloyd Murphy - LDM.B4.060 - B
107.55
106.88
77.18
76.95
75.24
73.53
70.18
69.83
69.31
65.51
64.97
63.64
39.52 DMSO-d6
20.76
20.25
f1 (ppm)

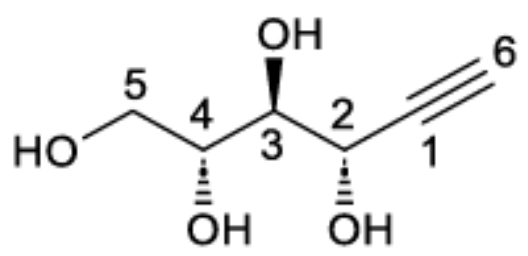
OH
6
5
4
3
2
HO
1
OH
OH

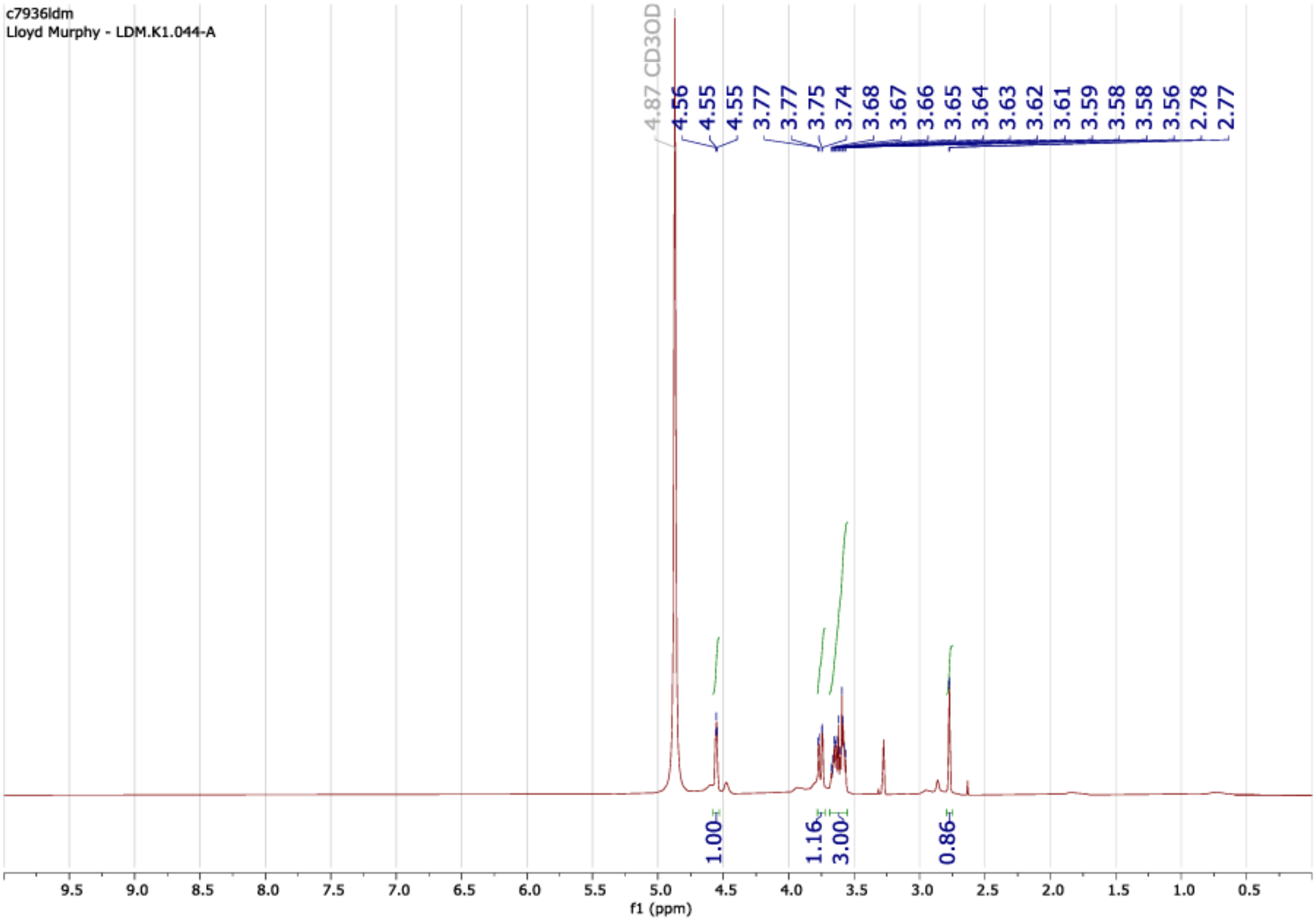
c7936ldm
Lloyd Murphy - LDM.K1.044-A
4.87 CD3OD
4.56
4.55
4.55
3.77
3.77
3.75
3.74
3.68
3.67
3.66
3.65
3.64
3.63
3.62
3.61
3.59
3.58
3.58
3.56
2.78
2.77
1.00
1.16
3.00
0.86
9.5
9.0
8.5
8.0
7.5
7.0
6.5
6.0
5.5
5.0
4.5
4.0
3.5
3.0
2.5
2.0
1.5
1.0
0.5
f1 (ppm)

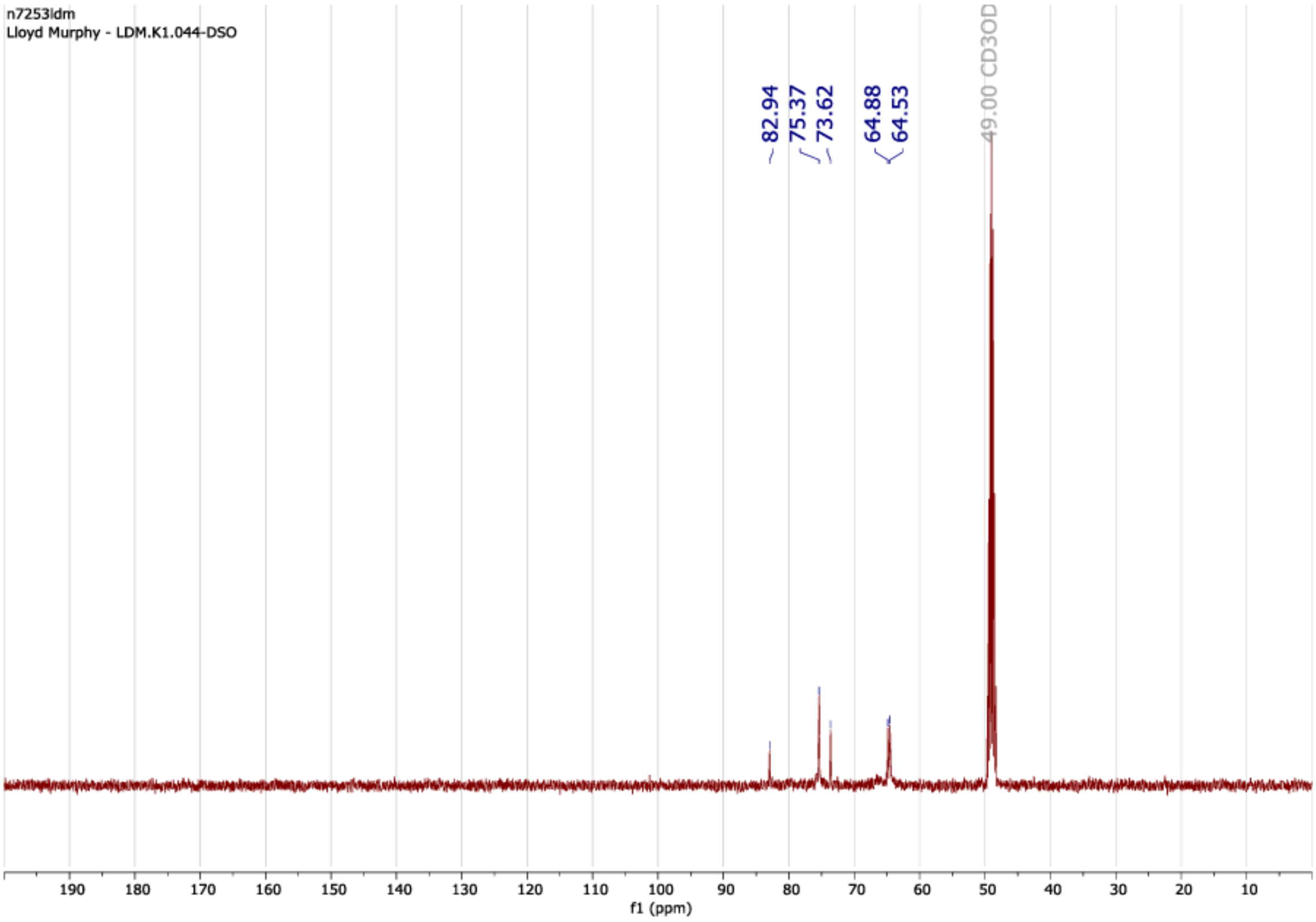
n7253ldm
Lloyd Murphy - LDM.K1.044-DSO
82.94
75.37
73.62
64.88
64.53
49.00 CD3OD
190
180
170
160
150
140
130
120
110
100
90
80
70
60
50
40
30
20
10
f1 (ppm)
